# Optimal Design of Reinforced Concrete Materials in Construction

**DOI:** 10.3390/ma15072625

**Published:** 2022-04-02

**Authors:** Mohammed Rady, Sameh Youssef Mahfouz, Salah El-Din Fahmy Taher

**Affiliations:** 1 Construction and Building Engineering Department, College of Engineering and Technology, Arab Academy for Science, Technology and Maritime Transport (AASTMT), B 2401 Smart Village, Giza 12577, Egypt; symahfouz@aast.edu; 2 Department of Structural Engineering, Faculty of Engineering, Tanta University, Tanta 31527, Egypt; salah.taher@f-eng.tanta.edu.eg

**Keywords:** construction materials, excel solver, evolutionary, structural design, solid slabs, flat slabs

## Abstract

The structural design process is iterative and involves many design parameters. Thus, this paper presents a controlled framework for selecting the adequate structural floor system for reinforced concrete buildings and efficiently utilizing the corresponding construction materials. Optimization was performed using an evolutionary algorithm to minimize the total construction cost, considering the costs of concrete, steel reinforcement, formwork, and labor. In the problem formulation, the characteristic compressive strength of concrete was treated as a design variable because it affects the mechanical performance of concrete. The design variables included the column spacings, concrete dimensions, and steel reinforcement of different structural components. The constraints reflected the Egyptian code of practice provisions. Because the choice of the structural floor system affects the design details, three systems were considered: solid slabs, flat slabs with drop panels, and flat slabs without drop panels. Two benchmark examples were presented, and the optimal design results of the structural floor systems were compared. The solid slab system had the lowest construction cost among the three structural floor systems. Comparative diagrams were developed to investigate the distribution of construction costs of each floor system. The results revealed that an adequate choice of design variables could save up to 17% of the building’s total construction cost.

## 1. Introduction

Reinforced concrete (RC) has been extensively used in building construction for its versatility and ease of construction [1]. The first step in any structural design process is choosing a proper structural system. This step is followed by specifying a concrete grade and determining preliminary concrete dimensions of the structural elements. Then, structural analysis is performed. The required steel reinforcement is then calculated to enhance the mechanical performance of structural elements such as ductility, tensile strength, and creep resistance [2,3,4,5,6,7]. Finally, the ultimate limit state and serviceability limit state requirements provided by design codes are checked [8,9,10,11]. If any design provision is not satisfied, the concrete dimensions should be adjusted, and accordingly, re-analysis of the structure is performed [12]. From the steps mentioned above, it can be concluded that the design problem involves many dependent and independent variables; therefore, the design process is iterative and time-consuming [13]. For these reasons, researchers have been exploring different optimization techniques to reduce the computational time and minimize the overall construction cost [12,13,14,15]. The optimization techniques investigated include the firefly algorithm [15], simulated annealing [13,16,17], and genetic algorithm [18,19,20]. Several studies used different algorithms built in the solver tool provided by Microsoft Excel [12,21,22].

The majority of previous studies focused on the optimization of individual RC structural elements, such as slabs [13,23,24], beams [12,18,22], columns [15,16,25], footings [21,26,27], and retaining walls [19,28,29]. The design procedures in these studies were subjected to specific code restrictions, such as the American concrete institute (ACI 318-19, ACI 318-14, ACI 318-05), Indian standards (IS: 456), Eurocode 2 (EN 1992-1-1:2004/A1:2014), and Brazilian standards (ABNT NBR 6118:2014, ABNT NBR 6118:2007). The design variables were limited to the structural element’s concrete dimensions and steel reinforcement. Several studies performed design optimization of RC three-dimensional large-scale structures with a single structural floor system [30,31,32,33]. Sahab et al. [30] performed cost optimization of buildings with flat slabs without drop panels, and they determined the optimal concrete dimensions and steel reinforcement of floors and columns. They also examined the effects of the column spacings on the optimal construction costs and found significant cost savings when the most economic column spacing was considered. Ženíšek et al. [31] studied the effects of ten concrete grades on the optimal construction costs of load-bearing structures. They investigated the effects of column spacings on the optimal results by considering two span variants (4 m and 8 m). Dehnavipour et al. [32] performed cost optimization of multi-story frames and determined the optimal concrete dimensions and steel reinforcement of beams and columns. Boscardin et al. [33] developed an optimization model to obtain the minimum construction cost of multi-story frames considering the steel reinforcement of columns and the concrete dimensions of beams and columns as design variables. The model was designed to investigate the effects of this automation on the optimal results. In another attempt, Robati et al. [34] conventionally designed a multi-story building by considering two structural floor systems and two types of concrete: flat slab with normal weight concrete, flat slab with ultra-lightweight concrete, waffle slab with normal weight concrete, and waffle slab with ultra-lightweight concrete. The authors found that the most economical design alternative was the waffle slab with normal weight concrete, which saved up to 7% on material consumption.

**Figure 1:**
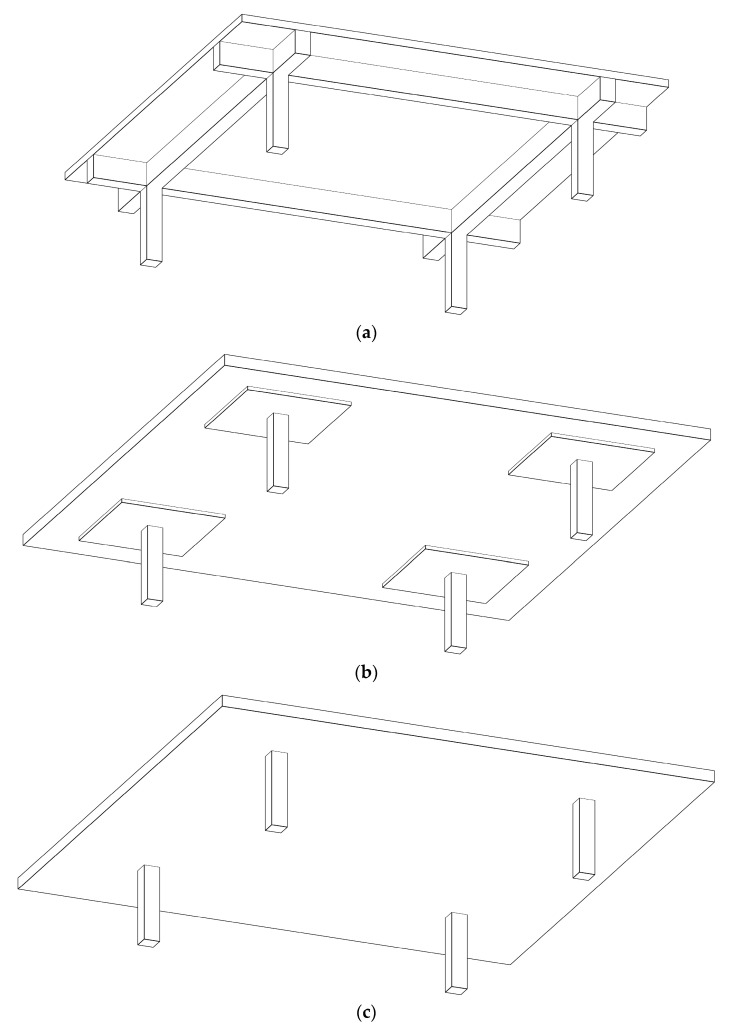
Building schemes for different structural floor systems considered in the current study: (**a**) solid slabs (SS); (**b**) flat slabs with drop panels (FSDP); (**c**) flat slabs without drop panels (FS).

Despite the intensive efforts of the researchers to minimize the costs of RC buildings, little attention was paid to considering different structural floor systems. Thus, the main aim of the current study was to determine the most economical structural floor system. To this end, three different structural floor systems were considered: solid slabs (SS), flat slabs with drop panels (FSDP), and flat slabs without drop panels (FS). Figure 1 provides a scheme of the building for each structural floor system. The structural components of each system were designed in accordance with the Egyptian code of practice for the design and construction of concrete structures (ECP 203-18) [35].

The design variables in most studies were limited to the concrete dimensions and steel reinforcement, neglecting the concrete characteristic compressive strength $f_{cu}$ and column spacings. The authors considered $f_{cu}$ as a design variable because it influences the mechanical performance of RC elements (i.e., elastic modulus, flexural strength, shear strength, punching strength, etc.). Accordingly, the concrete dimensions of structural components are dependent on $f_{cu}$. As $f_{cu}$ increases, the unit price of concrete $U_{c}$ increases and, consequently, the construction cost increases. Likewise, the column spacings were considered as design variables because they affect the straining actions (i.e., axial compressive loads, bending moments, shear loads), deflections, and choice of the concrete dimensions of the structural components. The design variables also included the optimal concrete dimensions and steel reinforcement of structural components. The objective function of the optimization problem was expressed in a mathematical formula, where the minimum construction cost of the floors and columns was sought. The construction cost of foundations was not included in the problem formulation because it is greatly dependent on the geotechnical properties of the soil. The solver tool provided by Microsoft Excel was used to perform the cost optimization. Comparative diagrams were developed to compare the optimal costs of construction materials and labor for different structural floor systems.

## 2. Structural Design

The structural design started with calculating the applied loads. These loads were calculated as per the Egyptian code for calculating loads and forces in structural and building works (ECL) [36]. Then, structural analysis was performed to obtain the straining actions. Finally, the concrete dimensions and steel reinforcement were determined to fulfill the design requirements. The design procedures of the slabs, beams, and columns are summarized in the following subsections.

### 2.1. RC Slabs

**Figure 2:**
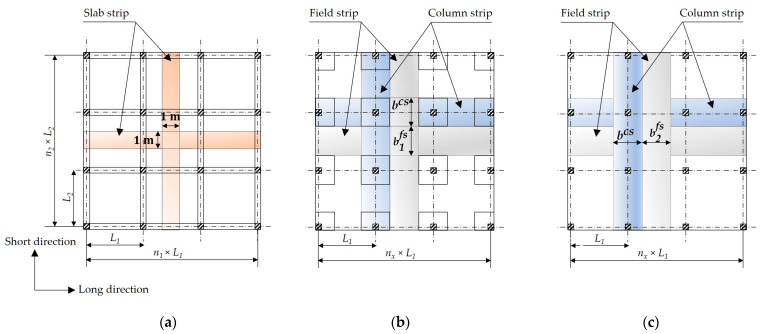
Analyzed strips of slabs for each structural floor system: (**a**) SS; (**b**) FSDP; (**c**) FS.

In the current study, the structural analysis of solid slabs and flat slabs was performed using the empirical methods provided by ECP 203-18 [35]. In the case of solid slabs, a strip of 1 m width was analyzed in the long and short directions. In the case of flat slabs, a column strip (a strip in the region of columns) and a field strip (a middle strip between the column strips) were analyzed in the long and short directions. Figure 2 shows the analyzed strips of slabs for each structural floor system. The design steps can be summarized as follows.

**Step 1.** Checking the rectangularity of the slabs as per Equation (1).

(1) $$\frac{L_{1}}{L_{2}}\;\leq \;\{\begin{array}{ll} 2\;\;\;\;\;\;\;\;\;\; & \mathrm{for}\;\mathrm{SS}\\ 1.33 & \mathrm{for}\;\mathrm{FSDP}\;\mathrm{and}\;\mathrm{FS} \end{array}\;$$

where $L_{1}$ and $L_{2}$ are the column spacings in the long and short directions, respectively.

**Step 2.** For flat slabs, calculation of the column strip width $b^{\mathrm{cs}}$, the field strip width in the long direction $b_{1}^{\mathrm{fs}}$, and the field strip width in the short direction $b_{2}^{\mathrm{fs}}$ using Equations (2)–(4), respectively.

(2) $$b^{\mathrm{cs}}=\{\begin{array}{l} S^{\mathrm{drop}}\;\mathrm{for}\;\mathrm{FSDP}\\ \frac{L_{2}}{2}\;\;\;\;\mathrm{for}\;\mathrm{FS} \end{array}$$

(3) $$b_{1}^{\mathrm{fs}}{=L}_{2}{\;-\;b}^{\mathrm{cs}}\;\mathrm{for}\;\mathrm{long}\;\mathrm{direction}$$

(4) $$b_{2}^{\mathrm{fs}}{=L}_{1}{\;-\;b}^{\mathrm{cs}}\;\mathrm{for}\;\mathrm{short}\;\mathrm{direction}$$

**Step 3.** Determining the preliminary slab thickness $t^{\mathrm{sl}}$ using Equation (5).

(5) $$t^{\mathrm{sl}}\;\geq \;\{\begin{array}{l} \max\left(\frac{L_{2}}{40},\;80\;\mathrm{mm}\right)\;\;\;\mathrm{for}\;\mathrm{SS}\\ \max\left(\frac{L_{1}}{36},\;150\;\mathrm{mm}\right)\;\mathrm{for}\;\mathrm{FSDP}\\ \max\left(\frac{L_{1}}{32},\;150\;\mathrm{mm}\right)\;\mathrm{for}\;\mathrm{FS} \end{array}\;$$

**Step 4.** For flat slabs with drop panels, determining the preliminary drop panel thickness $t^{\mathrm{drop}}$ using Equation (6) and the preliminary drop panel square width $S^{\mathrm{drop}}$ using Equation (7).

(6) $$t^{\mathrm{drop}}\;\geq \frac{\;t^{\mathrm{sl}}}{4}$$

(7) $$\frac{L_{1}}{3}\;\leq {\;S}^{\mathrm{drop}}\;\leq \;\frac{L_{2}}{2}\;$$

**Step 5.** Determining the preliminary width $b^{\mathrm{cl}}$ of each column utilizing Equation (8).

(8) $$b^{\mathrm{cl}}\geq \;\{\begin{array}{ll} 250\;\mathrm{mm};\;\mathrm{cl}=1,\;2,\;\ldots {,\;n}_{\mathrm{cl}} & \;\mathrm{for}\;\mathrm{SS}\\ \max\left(\frac{h}{15},\frac{L_{1}}{20},\;300\;\mathrm{mm}\;\right);\;\mathrm{cl}=1,\;2,\;\ldots {,\;n}_{\mathrm{cl}} & \mathrm{for}\;\mathrm{FSDP}\;\mathrm{and}\;\mathrm{FS} \end{array}$$

where *cl* represents the column under consideration and $n_{\mathrm{cl}}$ is the number of columns in a typical story.

**Step 6.** Calculation of the uniform load $w^{\mathrm{sl}}$ applied to the slabs utilizing Equation (9); this depends on the dead load *g* and the live load *p*. The dead load *g* is calculated using Equation (10), while the live load *p* is provided using ECL based on the function of the building [36].

(9) $$w^{\mathrm{sl}}=1.4g+1.6p$$

(10) $$g=\{\begin{array}{ll} \gamma_{\mathrm{rc}}t^{\mathrm{sl}}{+w}_{f} & \;\mathrm{for}\;\mathrm{SS}\\ \gamma_{\mathrm{rc}}t^{\mathrm{sl}}{+\gamma }_{\mathrm{rc}}\frac{t^{\mathrm{drop}}{\left(S^{\mathrm{drop}}\right)}^{2}}{L_{1}L_{2}}{+w}_{f}{+w}_{\mathrm{wl}} & \;\mathrm{for}\;\mathrm{FSDP}\\ \gamma_{\mathrm{rc}}t^{\mathrm{sl}}{+w}_{f}{+w}_{wl} & \;\mathrm{for}\;\mathrm{FS} \end{array}$$

where $\gamma_{\mathrm{rc}}$ is the unit weight of reinforced concrete; $w_{f}$ is the flooring load; $w_{\mathrm{wl}}$ is the partition wall load. 

**Step 7.** Calculation of the applied bending moments $M_{s,1}^{\mathrm{sl}}$ and $M_{s,2}^{\mathrm{sl}}$ at each critical cross-section of the long and short directions, respectively, as per Equations (11) and (12).

(11) $$M_{s,1}^{sl}=\left\{ \begin{array}{ll} \frac{\beta^{sl}w^{sl}{\left(L_{1}\right)}^{2}}{f_{s}};s=1,2,\ldots ,n_{s} & \mathrm{for}\;\mathrm{SS}\;(\mathrm{slab}\;\mathrm{strip};\;\mathrm{long}\;\mathrm{direction})\\ \frac{C_{s}}{b^{cs}}\left(\frac{w^{sl}L_{2}}{8}\right){\left(L_{1}-\frac{2b^{cl}}{3}\right)}^{2};s=1,2,\ldots ,n_{s} & \mathrm{for}\;\mathrm{FSDP}\;\mathrm{and}\;\mathrm{FS}\;(\mathrm{column}\;\mathrm{strip};\;\mathrm{long}\;\mathrm{direction})\\ \frac{F_{s}}{b_{1}^{fs}}\left(\frac{w^{sl}L_{2}}{8}\right){\left(L_{1}-\frac{2b^{cl}}{3}\right)}^{2};s=1,2,\ldots ,n_{s} & \mathrm{for}\;\mathrm{FSDP}\;\mathrm{and}\;\mathrm{FS}\;(\mathrm{field}\;\mathrm{strip};\;\mathrm{long}\;\mathrm{direction}) \end{array}\right.$$

(12) $$M_{s,2}^{s1}=\left\{ \begin{array}{ll} \frac{\alpha^{sl}w^{sl}{\left(L_{2}\right)}^{2}}{f_{s}};s=1,2,\ldots ,n_{s} & \mathrm{for}\;\mathrm{SS}\;(\mathrm{slab}\;\mathrm{strip};\;\mathrm{short}\;\mathrm{direction})\\ \frac{C_{s}}{b^{cs}}\left(\frac{w^{sl}L_{1}}{8}\right){\left(L_{2}-\frac{2b^{cl}}{3}\right)}^{2};s=1,2,\ldots ,n_{s} & \mathrm{for}\;\mathrm{FSDP}\;\mathrm{and}\;\mathrm{FS}\;(\mathrm{column}\;\mathrm{strip};\;\mathrm{short}\;\mathrm{direction})\\ \frac{F_{s}}{b_{2}^{fs}}\left(\frac{w^{sl}L_{1}}{8}\right){\left(L_{2}-\frac{2b^{cl}}{3}\right)}^{2};s=1,2,\ldots ,n_{s} & \mathrm{for}\;\mathrm{FSDP}\;\mathrm{and}\;\mathrm{FS}\;(\mathrm{field}\;\mathrm{strip};\;\mathrm{short}\;\mathrm{direction}) \end{array}\right.$$

where $\alpha^{\mathrm{sl}}$ and $\beta^{\mathrm{sl}}$ are the coefficients obtained from ECP 203 based on the slab rectangularity; $f_{s}$ is a factor obtained from ECP 203-18 based on the location of the critical cross-section; *s* represents the critical cross-section under consideration; $n_{s}$ is the number of critical cross-sections; and $C_{s}$ and $F_{s}$ are percentages of the total bending moment for the column strip and field strips, respectively. These percentages are obtained from ECP 203-18, depending on the location of the critical cross-section.

**Step 8.** Calculation of the maximum permissible bending moment $M^{\mathrm{sl},\max}$ using Equation (13).

(13) $$M^{\mathrm{sl},\max}=\frac{R_{\max}f_{cu}b^{\mathrm{sl}}{\left(d^{\mathrm{sl}}\right)}^{2}}{\gamma_{c}}$$

where $R_{\max}$ is a factor obtained from ECP 203-18 and is based on the value of the yield strength of the longitudinal steel reinforcement $f_{y}$, $b^{\mathrm{sl}}$ is the width of the slab cross-section, $d^{\mathrm{sl}}$ is the effective depth of the slab, and $\gamma_{c}$ is the safety reduction factor for concrete obtained from ECP 203-18.

**Step 9.** Checking that $M_{s,1}^{\mathrm{sl}}$ and $M_{s,2}^{\mathrm{sl}}$ at each critical cross-section does not exceed $M^{\mathrm{sl},\max}$.

**Step 10.** Calculation of the required steel reinforcement area $A_{s}^{\mathrm{sl},r}$ for each critical cross-section in the long and short directions as per Equation (14).

(14) $$A_{s}^{\mathrm{sl},r}=\frac{\frac{f_{y}d^{\mathrm{sl}}}{\gamma_{s}}\;-\sqrt{{\left(\frac{f_{y}d^{\mathrm{sl}}}{\gamma_{s}}\right)}^{2}-\;3\left[\frac{{\left(f_{y}\right)}^{2}\gamma_{c}M_{s}^{\mathrm{sl}}}{{\left(\gamma_{s}\right)}^{2}f_{cu}b^{\mathrm{sl}}}\right]}}{1.5\left[\frac{{\left(f_{y}\right)}^{2}\gamma_{c}}{{\left(\gamma_{s}\right)}^{2}f_{cu}b^{\mathrm{sl}}}\right]}{;\;s=1,\;2,\;\ldots ,\;n}_{s}\;$$

where $\gamma_{s}$ is the safety reduction factor for the steel reinforcement obtained from ECP 203-18 and $M_{s}^{\mathrm{sl}}$ is the applied bending moment in the direction under consideration.

**Step 11.** Calculation of the minimum and maximum permitted areas of steel reinforcement $A^{\mathrm{sl},\min}$ and $A^{\mathrm{sl},\max}$, respectively, as per Equations (15) and (16).

(15) $$A^{\mathrm{sl},\min}=\max\left(\frac{0.6}{f_{y}}b^{\mathrm{sl}}d^{\mathrm{sl}},\;{0.0015b}^{\mathrm{sl}}t^{\mathrm{sl}}\right)\;$$

(16) $$A^{\mathrm{sl},\max}=\frac{\left(\frac{{0.67f}_{cu}}{\gamma_{c}}\right)\left(1.25\frac{c_{\max}}{d}\right)b^{\mathrm{sl}}d^{\mathrm{sl}}}{\left(\frac{f_{y}}{\gamma_{s}}\right)}$$

where $c_{\max}$ is the maximum permitted distance between the neutral axis and extreme compression fibers. The ratio $\frac{c_{\max}}{d}$ is obtained from ECP 203-18 and is based on the value of $f_{y}$.

**Step 12.** Selecting the bar diameter $\phi_{s}^{\mathrm{sl}}$ for each critical cross-section in the long and short directions, which shall be greater than or equal to 10 mm. This step is followed by determining the number of bars $n_{s}^{\mathrm{sl}}$ per meter for each critical cross-section in the long and short directions as per Equation (17).

(17) $$5\;\leq {\;n}_{s}^{\mathrm{sl}}\;\leq {\;10;\;s=1,\;2,\;\ldots ,\;n}_{s}\;$$

**Step 13.** Calculation of the chosen steel reinforcement area $A_{s}^{\mathrm{sl},\mathrm{ch}}$ for each critical cross-section in the long and short directions.

**Step 14.** Checking that $A_{s}^{\mathrm{sl},\mathrm{ch}}$ satisfies the limits provided by Equation (18).

(18) $$\max\left(A_{s}^{\mathrm{sl},r}{,\;A}^{\mathrm{sl},\min}\right)\;\leq {\;A}_{s}^{\mathrm{sl},\mathrm{ch}}\;\leq {\;A}^{\mathrm{sl},\max}{;\;s=1,\;2,\;\ldots ,\;n}_{s}\;$$

**Step 15.** Determining the shrinkage steel reinforcement area $A^{\mathrm{sl},\mathrm{sh}}$ in each direction that satisfies the temperature and cracking requirements utilizing Equation (19).

(19) $$A^{\mathrm{sl},\mathrm{sh}}=\{\begin{array}{ll} 0 & \mathrm{if}\;t^{\mathrm{sl}}\;<\;160\;\mathrm{mm}\\ \max\left({0.02A}^{\mathrm{sl},m},\;{392.7\;\mathrm{mm}}^{2}\right) & \mathrm{if}\;t^{\mathrm{sl}}\;\geq \;160\;\mathrm{mm} \end{array}\;$$

where $A^{\mathrm{sl},m}$ is the area of the maximum bottom steel reinforcement in each direction.

**Step 16.** Calculation of the tensile strength of concrete $f_{\mathrm{ctr}}$ using Equation (20).

(20) $$f_{\mathrm{ctr}}=0.6\sqrt{f_{cu}}\;$$

**Step 17.** Calculation of the cracking bending moment $M^{\mathrm{sl},\mathrm{cr}}$ as per Equation (21).

(21) $$M^{\mathrm{sl},\mathrm{cr}}=\frac{f_{\mathrm{ctr}}I^{\mathrm{sl},g}}{y^{\mathrm{sl}}}\;$$

where $I^{\mathrm{sl},g}$ is the gross moment of inertia of the slab cross-section, neglecting the cross-sectional area of steel reinforcement, and $y^{\mathrm{sl}}$ is the distance from the neutral axis to the extreme tension fibers of the slab gross cross-section.

**Step 18.** Calculation of the unfactored applied bending moments $M_{s}^{\mathrm{sl},d}$, $M_{s}^{\mathrm{sl},l}$, and $M_{s}^{\mathrm{sl},t}$ resulting from the dead, live, and total loads, respectively, as per Equations (22)–(24), respectively.

(22) $$M_{s}^{\mathrm{sl},d}=\{\begin{array}{l} \frac{\beta^{\mathrm{sl}}g{\left(L_{2}\right)}^{2}}{f_{s}};\;s=1,\;2,\;\ldots {,\;n}_{s}\;\;\;\;\;\;\;\;\;\;\;\;\;\;\;\;\;\;\;\;\;\;\;\;\;\;\mathrm{for}\;\mathrm{SS}\\ \frac{C_{s}}{b^{\mathrm{cs}}}\left(\frac{{\mathrm{gL}}_{2}}{8}\right){\left(L_{1}-\;\frac{{2b}^{\mathrm{cl}}}{3}\right)}^{2};\;s=1,\;2,\;\ldots {,\;n}_{s}\;\mathrm{for}\;\mathrm{FSDP}\;\mathrm{and}\;\mathrm{FS} \end{array}\;\;$$

(23) $$M_{s}^{\mathrm{sl},l}=\{\begin{array}{l} \frac{\beta^{\mathrm{sl}}p{\left(L_{2}\right)}^{2}}{f_{s}};\;s=1,\;2,\;\ldots {,\;n}_{s}\;\;\;\;\;\;\;\;\;\;\;\;\;\;\;\;\;\;\;\;\;\;\;\;\;\;\mathrm{for}\;\mathrm{SS}\\ \frac{C_{s}}{b^{\mathrm{cs}}}\left(\frac{{\mathrm{pL}}_{2}}{8}\right){\left(L_{1}-\;\frac{{2b}^{\mathrm{cl}}}{3}\right)}^{2};\;s=1,\;2,\;\ldots {,\;n}_{s}\;\mathrm{for}\;\mathrm{FSDP}\;\mathrm{and}\;\mathrm{FS} \end{array}\;$$

(24) $$M_{s}^{\mathrm{sl},t}{=M}_{s}^{\mathrm{sl},d}{+M}_{s}^{\mathrm{sl},l};\;s=1,\;2,\;\ldots {,\;n}_{s}$$

**Step 19.** Calculation of the elastic modulus of concrete $E_{c}$ as per Equation (25).

(25) $$E_{c}=4400\sqrt{f_{cu}}$$

**Step 20.** Determining the distance $z^{\mathrm{sl}}$ from the neutral axis and the extreme compression fibers of the slab cracked cross-section at the midspan using Equation (26).

(26) $$z^{\mathrm{sl}}=\;\{\begin{array}{l} \frac{{-\mathrm{nA}}_{2}^{\mathrm{sl},b}+\sqrt{{\left({\mathrm{nA}}_{2}^{\mathrm{sl},b}\right)}^{2}{+2\mathrm{nb}}^{\mathrm{sl}}A_{2}^{\mathrm{sl},b}d^{\mathrm{sl}}}}{b^{\mathrm{sl}}}\;\mathrm{for}\;\mathrm{SS}\\ \frac{{-\mathrm{nA}}_{1}^{\mathrm{sl},b}+\sqrt{{\left({\mathrm{nA}}_{1}^{\mathrm{sl},b}\right)}^{2}{+2\mathrm{nb}}^{\mathrm{sl}}A_{1}^{\mathrm{sl},b}d^{\mathrm{sl}}}}{b^{\mathrm{sl}}}\;\mathrm{for}\;\mathrm{FSDP}\;\mathrm{and}\;\mathrm{FS} \end{array}\;$$

where *n* is the modular ratio of concrete to steel, and $A_{1}^{\mathrm{sl},b}$ and $A_{2}^{\mathrm{sl},b}$ are the chosen areas of the maximum bottom reinforcement in the long and short directions, respectively. 

**Figure 3:**
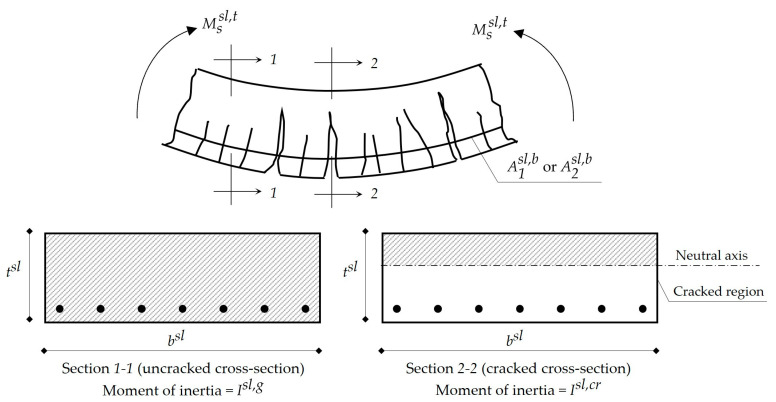
Cracking modes of slabs against applied bending moments.

**Step 21.** Calculation of the moment of inertia $I^{\mathrm{sl},\mathrm{cr}}$ of the slab cracked cross-section at the midspan as per Equation (27). Figure 3 illustrates the cracking modes of slabs against applied bending moments.

(27) $$I^{\mathrm{sl},\mathrm{cr}}=\{\begin{array}{l} \frac{b^{\mathrm{sl}}{\left(z^{\mathrm{sl}}\right)}^{3}}{3}{+\mathrm{nA}}_{2}^{\mathrm{sl},b}{\left(d^{\mathrm{sl}}{\;-\;z}^{\mathrm{sl}}\right)}^{2}\;\mathrm{for}\;\mathrm{SS}\\ \frac{b^{\mathrm{sl}}{\left(z^{\mathrm{sl}}\right)}^{3}}{3}{+\mathrm{nA}}_{1}^{\mathrm{sl},b}{\left(d^{\mathrm{sl}}{\;-\;z}^{\mathrm{sl}}\right)}^{2}\;\mathrm{for}\;\mathrm{FSDP}\;\mathrm{and}\;\mathrm{FS} \end{array}\;$$

**Step 22.** Calculation of the effective moment of inertia $I^{\mathrm{sl},e}$ of the slab cross-section at the midspan as per Equation (28).

(28) $$I^{\mathrm{sl},e}=\{\begin{array}{l} I^{\mathrm{sl},g}{\;\;\;\;\;\;\;\;\;\;\;\;\;\;\;\;\;\;\;\;\;\;\;\;\;\;\;\;\;\;\;\;\;\;\;\;\;\;\;\;\;\;\;\;\;\;\;\;\;\;\;\;\;\;\;\;\;\;\;\;\mathrm{if}\;M}^{\mathrm{sl},\mathrm{tmid}}\;\leq {\;M}^{\mathrm{sl},\mathrm{cr}}\\ I^{\mathrm{sl},g}{\left(\frac{M^{\mathrm{sl},\mathrm{cr}}}{M^{\mathrm{sl},\mathrm{tmid}}}\right)}^{3}{+I}^{\mathrm{sl},\mathrm{cr}}\left[1\;-\;{\left(\frac{M^{\mathrm{sl},\mathrm{cr}}}{M^{\mathrm{sl},\mathrm{tmid}}}\right)}^{3}\right]{\;\mathrm{if}\;M}^{\mathrm{sl},\mathrm{tmid}}\;\geq {\;M}^{\mathrm{sl},\mathrm{cr}} \end{array}\;$$

where $M^{\mathrm{sl},\mathrm{tmid}}$ is the unfactored applied bending moment resulting from the total load at the midspan.

**Step 23.** Calculation of the short-term deflections $\Delta^{\mathrm{sl},d}$, $\Delta^{\mathrm{sl},l}$, and $\Delta^{\mathrm{sl},\mathrm{st}}$ at the midspan resulting from the dead, live, and total loads, respectively, as per Equations (29)–(31).

(29) $$\Delta^{\mathrm{sl},d}=\{\begin{array}{l} \frac{5{\left(L_{2}\right)}^{2}}{{48E}_{c}I^{\mathrm{sl},e}}\left[M^{\mathrm{sl},\mathrm{dmid}}\;-\;0.1\left(M^{\mathrm{sl},\mathrm{dleft}}{+M}^{\mathrm{sl},\mathrm{dright}}\right)\right]\;\mathrm{for}\;\mathrm{SS}\\ \frac{5{\left(L_{1}\right)}^{2}}{{48E}_{c}I^{\mathrm{sl},e}}\left[M^{\mathrm{sl},\mathrm{dmid}}\;-0.1\left(M^{\mathrm{sl},\mathrm{dleft}}{+M}^{\mathrm{sl},\mathrm{dright}}\right)\right]\;\mathrm{for}\;\mathrm{FSDP}\;\mathrm{and}\;\mathrm{FS} \end{array}\;$$

(30) $$\Delta^{\mathrm{sl},l}=\Delta^{\mathrm{sl},d}\left(\frac{p}{g}\right)$$

(31) $$\Delta^{\mathrm{sl},\mathrm{st}}=\Delta^{\mathrm{sl},d}+\Delta^{\mathrm{sl},l}\;$$

where $M^{\mathrm{sl},\mathrm{dmid}}$, $M^{\mathrm{sl},\mathrm{dleft}}$, and $M^{\mathrm{sl},\mathrm{dright}}$ are the unfactored applied bending moments resulting from the dead load at the midspan, left support, and right support, respectively.

**Step 24.** Calculation of the total long-term deflection $\Delta^{\mathrm{sl},\mathrm{lt}}$ at the midspan utilizing Equation (32).

(32) $$\Delta^{\mathrm{sl},\mathrm{lt}}=\Delta^{\mathrm{sl},\mathrm{st}}{+\alpha }^{\mathrm{lt}}\Delta^{\mathrm{sl},d}\;$$

where $\alpha^{\mathrm{lt}}$ is a factor obtained from ECP 203-18 to consider the effects of creep.

**Step 25.** Checking that $\Delta^{\mathrm{sl},\mathrm{lt}}$ does not exceed the permitted deflection imposed by ECP 203-18 as per Equation (33).

(33) $$\Delta^{\mathrm{sl},\mathrm{lt}}\;\leq \;\{\begin{array}{l} \frac{L_{2}}{250}\;\mathrm{for}\;\mathrm{SS}\\ \frac{L_{1}}{250}\;\mathrm{for}\;\mathrm{FSDP}\;\mathrm{and}\;\mathrm{FS} \end{array}\;$$

**Step 26.** Check the punching shear stresses, where this shall be applied for flat slabs only because the presence of beams eliminates the risk of punching shear failure. The check starts with determining the effective depth $d_{p}$ which resists the punching shear stresses as per Equation (34).

(34) $$d_{p}=\{\begin{array}{l} d^{\mathrm{sl}}{+t}^{\mathrm{drop}}\;\mathrm{for}\;\mathrm{FSDP}\\ d^{\mathrm{sl}}\;\;\;\;\;\;\;\;\;\;\;\;\mathrm{for}\;\mathrm{FS} \end{array}\;$$

**Step 27.** For each slab–column connection, calculation of the critical shear perimeter $p^{\mathrm{cl}}$, the critical shear area $A^{\mathrm{cl},\mathrm{cr}}$, and the tributary area $A^{\mathrm{cl},\mathrm{tr}}$. These calculations are presented in Table 1, where the critical cross-section for punching shear is at a distance 0.5$d_{p}$ from the column face.

**Step 28.** Calculation of the applied punching shear load $Q^{\mathrm{cl}}$ as per Equation (35).

(35) $$Q^{\mathrm{cl}}{=w}^{\mathrm{sl}}\left(A^{\mathrm{cl},\mathrm{tr}}{\;-\;A}^{\mathrm{cl},\mathrm{cr}}\right){;\;\mathrm{cl}=1,\;2,\;\ldots ,\;n}_{\mathrm{cl}}\;\;$$

**Step 29.** Calculation of the applied punching shear stress $q^{\mathrm{cl}}$ using Equation (36).

(36) $$q^{\mathrm{cl}}=\frac{Q^{\mathrm{cl}}\beta^{\mathrm{cl}}}{p^{\mathrm{cl}}d_{p}}{;\;\mathrm{cl}=1,\;2,\;\ldots ,\;n}_{\mathrm{cl}}$$

where $\beta^{\mathrm{cl}}$ is a factor obtained from ECP 203-18 based on the column location.

**Step 30.** Calculation of the nominal concrete punching shear strength $q^{\mathrm{cl},\max}$ utilizing Equation (37).

(37) $$q^{\mathrm{cl},\max}=\min\left[0.8\left(\frac{\alpha^{\mathrm{cl}}d_{p}}{p^{\mathrm{cl}}}+0.2\right)\sqrt{\frac{f_{cu}}{\gamma_{c}}},\;0.316\sqrt{\frac{f_{cu}}{\gamma_{c}}},\;1.7\;\mathrm{MPa}\right]{;\;\mathrm{cl}=1,\;2,\;\ldots ,\;n}_{\mathrm{cl}}$$

where $\alpha^{\mathrm{cl}}$ is a factor obtained from ECP 203-18 based on the column location.

**Step 31.** Checking that $q^{\mathrm{cl}}$ does not exceed the corresponding $q^{\mathrm{cl},\max}$.

### 2.2. RC Beams

**Figure 4:**
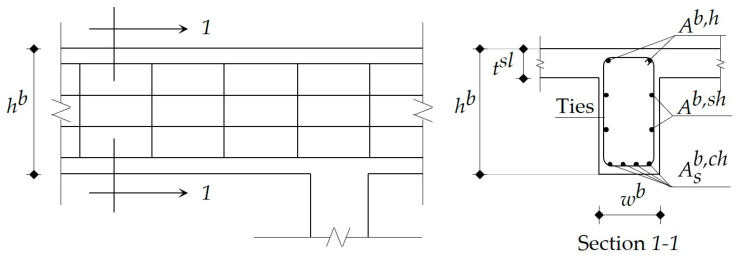
Typical arrangement of the steel reinforcement of a beam.

In the current study, the beams existed only in the solid slab structural floor system, and these were classified into four groups: interior beams in the long direction, interior beams in the short direction, edge beams in the long direction, and edge beams in the short direction. Using a conservative approach, all cross-sections of beams were designed as rectangular sections. Figure 4 illustrates the typical arrangement of the steel reinforcement of a beam. In practice, the concrete dimensions of all beams in a typical story are the same to attain simpler formwork, and these are reflected in the following design steps.

**Step 1.** Determining the preliminary beam height $h^{b}$ utilizing Equation (38).

(38) $$h^{b}\;\geq \;\max\left({3t}^{\mathrm{sl}},\;400\;\mathrm{mm}\right){;\;b=1,\;2,\;\ldots ,\;n}_{b}\;$$

where *b* represents the beam under consideration and $n_{b}$ is the number of beams in a typical story.

**Step 2.** Determining the preliminary beam width $w^{b}$ using Equation (39).

(39) $$w^{b}\;\geq \;\max\left(t_{w},\;250\;\mathrm{mm}\right){;\;b=1,\;2,\;\ldots ,\;n}_{b}\;$$

where $t_{w}$ is the partition wall thickness.

**Step 3.** Checking that the span-to-depth ratio $\left(\frac{L^{b}}{d^{b}}\right)$ of each beam is greater than or equal to 4, where $L^{b}$ is the beam span length and $d^{b}$ is the effective depth of the beam (the distance between the steel reinforcement and the extreme compression fibers of the beam).

**Step 4.** Checking the side buckling for each beam. This can be achieved when the span is less than or equal to the two values $\frac{200{\left(w^{b}\right)}^{2}}{d^{b}}$ and ${40w}^{b}$.

**Step 5.** Calculation of the uniform load applied on each beam $W^{b}$ as per Equation (40).

(40) $$W^{b}=1.4\left(\gamma_{\mathrm{rc}}w^{b}h^{b}\;{+w}_{\mathrm{wl}}\right){+N}^{b}\alpha^{b}w^{\mathrm{sl}}L_{2}{;\;b=1,\;2,\;\ldots ,\;n}_{b}\;$$

where $N^{b}$ is the number of slabs supported by the beam under consideration (i.e., $N^{b}$ equals 1 for edge beams and 2 for interior beams) and $\alpha^{b}$ is a coefficient obtained from ECP 203-18 based on the direction under consideration.

**Step 6.** Calculation of the applied bending moment $M_{s}^{b}$ at each critical cross-section of the beam under consideration as per Equation (41).

(41) $$M_{s}^{b}=\frac{W^{b}{\left(L^{b}\right)}^{2}}{h_{s}}{;\;s=1,\;2,\;\ldots ,\;n}_{s}{;\;b=1,\;2,\;\ldots ,\;n}_{b}$$

where $L^{b}$ is the beam span length, $h_{s}$ is a factor obtained from ECP 203-18 and is based on the location of the critical cross-section, and $n_{s}$ is the number of critical cross-sections of a beam.

**Step 7.** Calculation of the maximum permissible bending moment $M^{b,\max}$ for each beam utilizing Equation (42).

(42) $$M^{b,\max}=\frac{R_{\max}f_{\mathrm{cu}}W^{b}{\left(d^{b}\right)}^{2}}{\gamma_{c}}{;\;b=1,\;2,\;\ldots ,\;n}_{b}$$

**Step 8.** Checking that $M_{s}^{b}$ at each critical cross-section of each beam does not exceed the corresponding $M^{b,\max}$.

**Step 9.** Calculation of the required steel reinforcement area $A_{s}^{b,r}$ for each critical cross-section of each beam using Equation (43).

(43) $$A_{s}^{b,r}=\frac{\frac{f_{y}d^{b}}{\gamma_{s}}\;-\;\sqrt{{\left(\frac{f_{y}d^{b}}{\gamma_{s}}\right)}^{2}\;-\;3\left[\frac{{\left(f_{y}\right)}^{2}\gamma_{c}M_{s}^{b}}{{\left(\gamma_{s}\right)}^{2}f_{\mathrm{cu}}w^{b}}\right]}}{1.5\left[\frac{{\left(f_{y}\right)}^{2}\gamma_{c}}{{\left(\gamma_{s}\right)}^{2}f_{\mathrm{cu}}w^{b}}\right]}{;\;s=1,\;2,\;\ldots ,\;n}_{s}{;\;b=1,\;2,\;\ldots ,\;n}_{b}\;$$

**Step 10.** Calculation of the minimum and maximum permitted areas of steel reinforcement $A_{s}^{b,\min}$ and $A^{b,\max}$, respectively, for each critical cross-section of each beam as per Equations (44) and (45).

(44) $$A_{s}^{b,\min}=\min\left[{0.0015w}^{b}d^{b},\;\max\left(\frac{1.1}{f_{y}}w^{b}d^{b},\;\frac{0.225\sqrt{f_{\mathrm{cu}}}}{f_{y}}w^{b}d^{b},\;{1.3A}_{s}^{b,r}\right)\right];s=1,\;2,\;\ldots {,\;n}_{s}{;\;b=1,\;2,\;\ldots ,\;n}_{b}\;$$

(45) $$A^{b,\max}=\frac{\left(\frac{{0.67f}_{\mathrm{cu}}}{\gamma_{c}}\right)\left(1.25\frac{c_{\max}}{d^{b}}\right)w^{b}d^{b}}{\left(\frac{f_{y}}{\gamma_{s}}\right)}{;\;b=1,\;2,\;\ldots ,\;n}_{b}$$

**Step 11.** Determining the beam bar diameter $\phi^{b}$, where this shall be greater than or equal to 10 mm. This step is followed by determining the number of bars $n_{s}^{b}$ for each critical cross-section of each beam as per Equation (46).

(46) $$2\;\leq {\;n}_{s}^{b}\;\leq {\;12;\;s=1,\;2,\;\ldots ,\;n}_{s}{;\;b=1,\;2,\;\ldots ,\;n}_{b}\;$$

**Step 12.** Calculation of the chosen steel reinforcement area $A_{s}^{b,\mathrm{ch}}$ for each critical cross-section of each beam.

**Step 13.** Checking that $A_{s}^{b,\mathrm{ch}}$ for each critical cross-section of each beam satisfies the limits provided by Equation (47).

(47) $$\max\left(A_{s}^{b,r}{,\;A}_{s}^{b,\min}\right)\;\leq {\;A}_{s}^{b,\mathrm{ch}}\;\leq {\;A}^{b,\max}{;\;s=1,\;2,\;\ldots ,\;n}_{s}{;\;b=1,\;2,\;\ldots ,\;n}_{b}\;$$

**Step14.** Determining the shrinkage steel reinforcement area $A^{b,\mathrm{sh}}$ for each beam that satisfies the temperature and cracking requirements as per Equation (48).

(48) $$A^{b,sh}=\left\{ \begin{array}{ll} 0;b=1,2,\ldots ,n_{b} & \mathrm{if}\left(h^{b}-t^{sl}\right)\leq 600\;mm\\ 0.08A^{b,m};b=1,2,\ldots ,n_{b} & \mathrm{if}\left(h^{b}-t^{sl}\right)>600\;mm \end{array}\right.$$

where $A^{b,m}$ is the area of the maximum bottom steel reinforcement for each beam.

**Step15.** Determining the top steel reinforcement area $A^{b,h}$ required to hang the lateral ties for each beam as per Equation (49).

(49) $${0.1A}^{b,m}\;\leq {\;A}^{b,h}\;\leq \;{0.2A}^{b,m}{;\;b=1,\;2,\;\ldots ,\;n}_{b}$$

**Step 16.** Calculation of the cracking bending moment $M^{b,\mathrm{cr}}$ as per Equation (50).

(50) $$M^{b,\mathrm{cr}}=\frac{f_{\mathrm{ctr}}I^{b,g}}{y^{b}}{;\;b=1,\;2,\;\ldots ,\;n}_{b}\;$$

where $I^{b,g}$ is the gross moment of inertia of the beam cross-section, neglecting the cross-sectional area of steel reinforcement, and $y^{b}$ is the distance from the neutral axis to the extreme tension fibers of the beam gross cross-section.

**Step 17.** Calculation of the unfactored applied bending moments $M_{s}^{b,d}$, $M_{s}^{b,l}$, and $M_{s}^{b,t}$ resulting from the dead load, live load, and total load, respectively, at each critical cross-section of each beam using Equations (51)–(53).

(51) $$M_{s}^{b,d}=\frac{\left(\gamma_{\mathrm{rc}}b^{b}t^{b}{+w}_{\mathrm{wl}}{+N}^{b}\alpha^{b}{\mathrm{gL}}_{2}\right){\left(L^{b}\right)}^{2}}{h_{s}};s=1,\;2,\;\ldots {,\;n}_{s}{;\;b=1,\;2,\;\ldots ,\;n}_{b}\;$$

(52) $$M_{s}^{b,l}=\frac{\left(N^{b}\alpha^{b}{\mathrm{pL}}_{2}\right){\left(L^{b}\right)}^{2}}{h_{s}};s=1,\;2,\;\ldots {,\;n}_{s}{;\;b=1,\;2,\;\ldots ,\;n}_{b}$$

(53) $$M_{s}^{b,t}{=M}_{s}^{b,d}{+M}_{s}^{b,l};s=1,\;2,\;\ldots {,\;n}_{s}{;\;b=1,\;2,\;\ldots ,\;n}_{b}$$

**Step 18.** Determining the distance $z^{b}$ from the neutral axis and the extreme compression fibers of the cracked cross-section of each beam at the midspan using Equation (54).

(54) $$z^{b}=\;\frac{{-\mathrm{nA}}^{b,m}+\sqrt{{\left({\mathrm{nA}}^{b,m}\right)}^{2}{+2\mathrm{nw}}^{b}A^{b,m}d^{b}}}{w^{b}}{;\;b=1,\;2,\;\ldots ,\;n}_{b}\;$$

**Step 19.** Calculation of the cracked moment of inertia $I^{b,\mathrm{cr}}$ for each beam at the midspan as per Equation (55).

(55) $$I^{b,\mathrm{cr}}=\frac{w^{b}{\left(z^{b}\right)}^{3}}{3}{+\mathrm{nA}}^{b,m}{\left(d^{b}{\;-\;z}^{b}\right)}^{2}{;\;b=1,\;2,\;\ldots ,\;n}_{b}\;$$

**Step 20.** Calculation of the effective moment of inertia $I^{b,e}$ for each beam at the midspan as per Equation (56).

(56) $$I^{b,e}=\{\begin{array}{ll} I^{b,g}{;\;b=1,\;2,\;\ldots ,\;n}_{b} & {\;\mathrm{if}\;M}^{b,\mathrm{tmid}}\;\leq {\;M}^{b,\mathrm{cr}}\\ I^{b,g}{\left(\frac{M^{b,\mathrm{cr}}}{M^{b,\mathrm{tmid}}}\right)}^{3}{+I}^{b,\mathrm{cr}}\left[1\;-\;{\left(\frac{M^{b,\mathrm{cr}}}{M^{b,\mathrm{tmid}}}\right)}^{3}\right]{;\;b=1,\;2,\;\ldots ,\;n}_{b} & {\;\mathrm{if}\;M}^{b,\mathrm{tmid}}\;\geq {\;M}^{b,\mathrm{cr}} \end{array}\;$$

where $M^{b,\mathrm{tmid}}$ is the unfactored applied bending moment resulting from the total load at the midspan of each beam.

**Step 21.** Calculation of the short-term deflections $\Delta^{b,d}$, $\Delta^{b,l}$, and $\Delta^{b,\mathrm{st}}$ resulting from the dead load, live load, and total load, respectively, at the midspan of each beam using Equations (57)–(59), respectively.

(57) $$\Delta^{b,d}=\frac{{5L}^{2}}{{48E}_{c}I^{b,e}}\left[M^{b,\mathrm{dmid}}-0.1\left(M^{b,\mathrm{dleft}}{+M}^{b,\mathrm{dright}}\right)\right]{;\;b=1,\;2,\;\ldots ,\;n}_{b}\;$$

(58) $$\Delta^{b,l}=\frac{{5L}^{2}}{{48E}_{c}I^{b,e}}\left[M^{b,\mathrm{lmid}}-0.1\left(M^{b,\mathrm{lleft}}{+M}^{b,\mathrm{lright}}\right)\right]{;\;b=1,\;2,\;\ldots ,\;n}_{b}$$

(59) $$\Delta^{b,\mathrm{st}}=\Delta^{b,d}+\Delta^{b,l}{;\;b=1,\;2,\;\ldots ,\;n}_{b}\;$$

where $M^{b,\mathrm{dmid}}$, $M^{b,\mathrm{dleft}}$, and $M^{b,\mathrm{dright}}$ are the unfactored applied bending moments resulting from the dead load at the midspan, left support, and right support, respectively; $M^{b,\mathrm{lmid}}$, $M^{b,\mathrm{lleft}}$, and $M^{b,\mathrm{lright}}$ are the unfactored applied bending moments resulting from the live load at the midspan, left support, and right support, respectively.

**Step 22.** Calculation of the long-term deflection $\Delta^{b,\mathrm{lt}}$ at the midspan of each beam as per Equation (60).

(60) $$\Delta^{b,\mathrm{lt}}=\Delta^{b,\mathrm{st}}{+\alpha }^{\mathrm{lt}}\Delta^{b,d}{;\;b=1,\;2,\;\ldots ,\;n}_{b}\;$$

**Step 23.** Checking that $\Delta^{b,\mathrm{lt}}$ does not exceed the permitted deflection imposed by ECP 203-18 as per Equation (61).

(61) $$\Delta^{b,\mathrm{lt}}\;\leq \;\frac{L^{b}}{250}{;\;b=1,\;2,\;\ldots ,\;n}_{b}\;$$

**Step 24.** Calculation of the applied shear load $Q^{b}$ at the critical cross-section of each beam, i.e., at a distance 0.5$d^{b}$ from the corresponding column face using Equation (62).

(62) $$Q^{b}=W^{b}\left(1\;-\;\frac{b^{\mathrm{cl}}}{2}\;-\;\frac{d^{b}}{2}\right){;\;b=1,\;2,\;\ldots ,\;n}_{b}$$

**Step 25.** Calculation of the applied shear stress $q^{b}$ at the critical cross-section of each beam as per Equation (63).

(63) $$q^{b}=\frac{Q^{b}}{b^{b}d^{b}}{;\;b=1,\;2,\;\ldots ,\;n}_{b}$$

**Step 26.** Calculation of the maximum shear strength $q^{b,\max}$ using Equation (64).

(64) $$q^{\max}=\min\left(0.7\sqrt{\frac{f_{\mathrm{cu}}}{\gamma_{c}}},\;4.4\;\mathrm{MPa}\right)$$

**Step 27.** Checking that $q^{b}$ does not exceed $q^{\max}$.

**Step 28.** Calculation of the concrete nominal shear strengths $q^{\mathrm{uncr}}$ and $q^{\mathrm{cr}}$ in the uncracked and cracked stages, respectively, utilizing Equations (65) and (66).

(65) $$q^{\mathrm{uncr}}=0.16\sqrt{\frac{f_{\mathrm{cu}}}{\gamma_{c}}}$$

(66) $$q^{\mathrm{cr}}=0.12\sqrt{\frac{f_{\mathrm{cu}}}{\gamma_{c}}}$$

**Step 29.** Determining the shear stress $q^{b,t}$ of the vertical ties for each beam as per Equation (67).

(67) $$q^{b,t}=\{\begin{array}{l} {0;\;b=1,\;2,\;\ldots ,\;n}_{b}{\;\mathrm{if}\;q}^{b}\;\leq {\;q}^{\mathrm{uncr}}\\ q^{b}{\;-\;q}^{\mathrm{cr}}{;\;b=1,\;2,\;\ldots ,\;n}_{b}{\;\mathrm{if}\;q}^{b}{\;>\;q}^{\mathrm{uncr}} \end{array}\;$$

**Step 30.** Determining the number of branches $n^{b,t}$ of the vertical ties for each beam utilizing Equation (68).

(68) $$n^{b,t}\;\geq \;\{\begin{array}{l} {2;\;b=1,\;2,\;\ldots ,\;n}_{b}{\;\mathrm{if}\;w}^{b}\;<\;400\;\mathrm{mm}\\ {4;\;b=1,\;2,\;\ldots ,\;n}_{b}{\;\mathrm{if}\;w}^{b}\;\geq \;400\;\mathrm{mm} \end{array}\;$$

**Step 31.** Determining the spacing $S^{b,t}$ between vertical ties for each beam that satisfies the shear stress requirements provided by Equation (69).

(69) $$S^{b,t}=\{\begin{array}{ll} {200\;\mathrm{mm};\;b=1,\;2,\;\ldots ,\;n}_{b} & {\;\mathrm{if}\;q}^{b}\;\leq {\;q}^{\mathrm{uncr}}\\ \max\left(\frac{A^{b,t}n^{b,t}\left(f_{y}/\gamma_{s}\right)}{q^{b,t}b^{b}},\;200\;\mathrm{mm}\right){;\;b=1,\;2,\;\ldots ,\;n}_{b} & {\;\mathrm{if}\;q}^{b}{\;>\;q}^{\mathrm{cr}} \end{array}\;$$

where $A^{b,t}$ is the area of the vertical ties for each beam.

**Step 32.** Checking that $S^{b,t}$ is greater than or equal to 100 mm.

### 2.3. RC Columns

**Figure 5:**
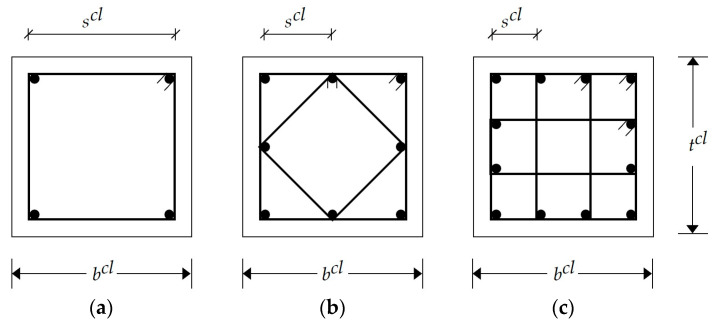
Possible steel reinforcement arrangements for columns: (**a**) 4 bars; (**b**) 8 bars; (**c**) 12 bars.

According to ECP 203-18, the columns should be designed to resist the axial loads and bending moments. In the current study, the columns were classified into four groups based on their locations: interior columns, edge columns in the x-direction, edge columns in the y-direction, and corner columns. For simplification, each group had a square cross-section. Three steel reinforcement arrangements were utilized to satisfy the design provisions (Figure 5). Each arrangement had a specific number of vertical bars and a shape of lateral ties based on the column width $b^{\mathrm{cl}}$. The design steps can be summarized as follows.

**Step 1.** Calculation of the concrete cross-sectional area $A^{\mathrm{cl}}$ of each column.

**Step 2.** Calculation of the axial design load $P^{\mathrm{cl}}$ for each column using Equation (70).

(70) $$P^{\mathrm{cl}}=\{\begin{array}{ll} N\left(r^{b}\overset{n_{b}}{\underset{b=1}{\sum }}R^{b,\mathrm{cl}}{+\gamma }_{\mathrm{rc}}A^{\mathrm{cl}}H\right){;\;\mathrm{cl}=1,\;2,\;\ldots ,\;n}_{\mathrm{cl}} & \;\mathrm{for}\;\mathrm{SS}\\ N\left(w^{\mathrm{sl}}A^{\mathrm{cl},\mathrm{tr}}{+\gamma }_{\mathrm{rc}}A^{\mathrm{cl}}H\right){;\;\mathrm{cl}=1,\;2,\;\ldots ,\;n}_{\mathrm{cl}} & \;\mathrm{for}\;\mathrm{FSDP}\;\mathrm{and}\;\mathrm{FS} \end{array}\;$$

where *N* is the number of stories, $r^{b}$ is a shear coefficient obtained from ECP 203-18 based on the column location, $R^{b,\mathrm{cl}}$ is the reaction of a beam at the beam–column connection under consideration, and *H* is the typical story height.

**Step 3.** Determining the steel arrangement of each column. The spacing $s^{\mathrm{cl}}$ between two adjacent bars shall not exceed 250 mm.

**Step 4.** Determining the bar diameter $\phi^{\mathrm{cl}}$ for each column, which shall be greater than or equal to 12 mm.

**Step 5.** Calculation of the chosen vertical steel reinforcement area $A^{\mathrm{cl},s}$ of each column.

**Step 6.** Checking that $A^{\mathrm{cl},s}$ of each column fulfills the reinforcement limits provided by Equation (71).

(71) $${0.008A}^{\mathrm{cl}}\;\leq {\;A}^{\mathrm{cl},s}\;\leq \;{0.04A}^{\mathrm{cl}}{;\;\mathrm{cl}=1,\;2,\;\ldots ,\;n}_{\mathrm{cl}}\;$$

**Step 7.** Determining the number of lateral ties $n^{\mathrm{cl},t}$ per meter for each column as per Equation (72).

(72) $$max\left(\frac{1000}{15\phi^{\mathrm{cl}}},\;5\right)\;\leq {\;n}^{\mathrm{cl},t}\;\leq {\;10;\;\mathrm{cl}=1,\;2,\;\ldots ,\;n}_{\mathrm{cl}}\;$$

**Step 8.** Calculation of the volume of steel lateral ties $V_{c}^{\;\mathrm{cl},t}$ per meter for each column.

**Step 9.** Checking that $V_{c}^{\;\mathrm{cl},t}$ for each column is greater than or equal to ${0.025A}^{\mathrm{cl}}$.

**Step 10.** Calculation of the maximum axial load $P^{\mathrm{cl},\max}$ that each column can withstand as per Equation (73).

(73) $$P^{\mathrm{cl},\max}={0.35f}_{\mathrm{cu}}{(A}^{\mathrm{cl}}{\;-\;A}^{\mathrm{cl},s})+{0.67f}_{y}A^{\mathrm{cl},s}{;\;\mathrm{cl}=1,\;2,\;\ldots ,\;n}_{\mathrm{cl}}$$

**Step 11.** Checking that the axial design load $P^{\mathrm{cl}}$ for each column does not exceed the corresponding $P^{\mathrm{cl},\max}$.

**Step 12.** For flat slabs only, calculation of the design bending moment $M^{\mathrm{cl}}$ transferred from the slabs to the columns utilizing Equation (74).

(74) $$M^{\mathrm{cl}}{=c}^{\mathrm{cl}}M_{s}^{\mathrm{sl}}{;\;\mathrm{cl}=1,\;2,\;\ldots ,\;n}_{\mathrm{cl}}$$

where $c^{\mathrm{cl}}$ is a factor obtained from ECP 203-18 based on the column location.

**Figure 6:**
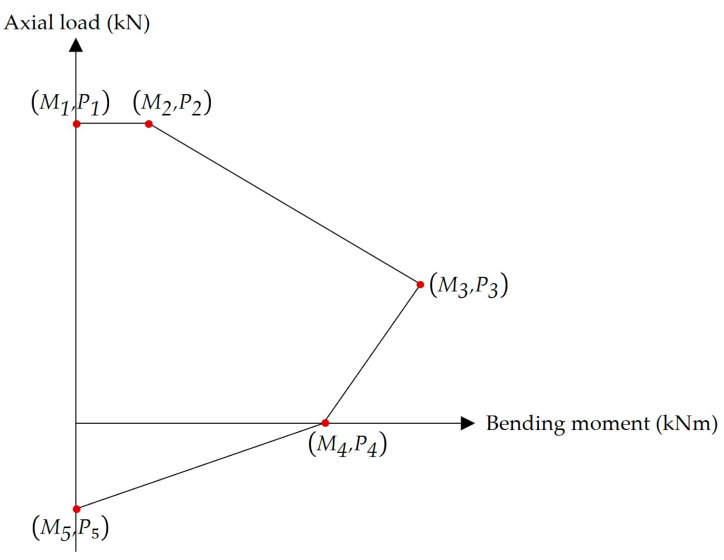
Typical interaction diagram for eccentrically loaded columns.

**Step 13.** For flat slabs only, constructing an interaction diagram for each column to check its safety. In this diagram, five points (i.e., combinations of axial loads and bending moments) were used to represent the failure envelope, i.e., the boundaries of the diagram (Figure 6).

1. Point 1 represents the pure axial compression failure mode. Here, the axial load $P_{1}$ equals $P^{\mathrm{cl},\max}$.
2. Point 2 represents the compression failure mode with minimum eccentricity. The bending moment $M_{2}$ can be calculated using Equation (75). The minimum eccentricity $e^{\mathrm{cl},\min}$ permitted by ECP 203-18 is expressed in Equation (76).
  (75) $$M_{2}{=P}^{\mathrm{cl},\max}\cdot e^{\mathrm{cl},\min}\;$$
  (76) $$e^{\mathrm{cl},\min}=\max\left({0.05t}^{\mathrm{cl}},\;20\;\mathrm{mm}\right)\;$$
3. Point 3 represents the balanced failure mode (i.e., the concrete failure and steel reinforcement yielding occur simultaneously). The axial load $P_{3}$ and the bending moment $M_{3}$ are expressed in Equations (77) and (78), respectively.
  (77) $$P_{3}=0.67\frac{f_{\mathrm{cu}}}{\gamma_{c}}a^{\mathrm{cl}}b^{\mathrm{cl}}+\frac{f_{y}}{\gamma_{s}}\left(A^{\mathrm{cl},s{}^{\prime }}{\;-\;A}^{\mathrm{cl},\mathrm{st}}\right)\;$$
  (78) $$M_{3}=0.67\frac{f_{\mathrm{cu}}}{\gamma_{c}}a^{\mathrm{cl}}b^{\mathrm{cl}}\left(X^{\mathrm{cl}}\;-\;\frac{a^{\mathrm{cl}}}{2}\right)+\frac{f_{y}}{\gamma_{s}}A^{\mathrm{cl},s{}^{\prime }}\left(X^{\mathrm{cl}}{\;-\;d}^{\mathrm{cl}{}^{\prime }}\right)+\frac{f_{y}}{\gamma_{s}}A^{\mathrm{cl},\mathrm{st}}\left(d^{\mathrm{cl}}{\;-\;X}^{\mathrm{cl}}\right)$$
  where $A^{\mathrm{cl},s{}^{\prime }}$ is the area of steel reinforcement on the compression side, $A^{\mathrm{cl},\mathrm{st}}$ is the area of steel reinforcement on the tension side, $a^{\mathrm{cl}}$ is the length of the equivalent rectangular stress block, $X^{\mathrm{cl}}$ is the distance from the plastic centroid to the extreme compression fibers of the column cross-section, $d^{\mathrm{cl}{}^{\prime }}$ is the distance from the center of compressive bars to the extreme compression fibers of the column cross-section, and $d^{\mathrm{cl}}$ is the distance from the center of tensile bars to the extreme compression fibers of the column cross-section.
4. Point 4 represents the pure bending failure mode. The bending moment $M_{4}$ can be calculated using Equation (79).
  (79) $$M_{4}{=f}_{y}A^{\mathrm{cl},\mathrm{st}}\left(d^{\mathrm{cl}}{\;-\;d}^{\mathrm{cl}{}^{\prime }}\right)$$
5. Point 5 represents the pure axial tension failure. The axial load $P_{5}$ can be calculated using Equation (80).
  (80) $$P_{5}{=f}_{y}A^{\mathrm{cl},s}\;$$

**Step 14.** For flat slabs only, checking the adequacy of each column. This step can be achieved by ensuring that the axial load $P^{\mathrm{cl}}$ and the bending moment $M^{\mathrm{cl}}$ for the column under consideration exist within the interaction diagram boundaries.

## 3. Construction Cost Parameters

The total cost of a construction project consists of a set of direct costs and indirect costs. The direct costs include materials and labor costs, while the indirect costs include accounting services, administration, and site overhead. The indirect costs are often independent of the design parameters. Thus, the current study considers the direct costs of the skeleton structure of the RC building. Here, the costs of concrete, steel reinforcement, formwork, and labor of floors and columns are considered.

**Figure 7:**
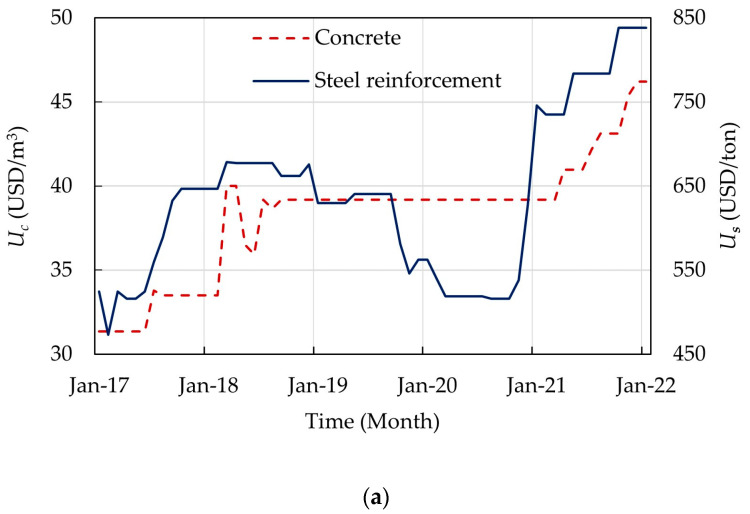

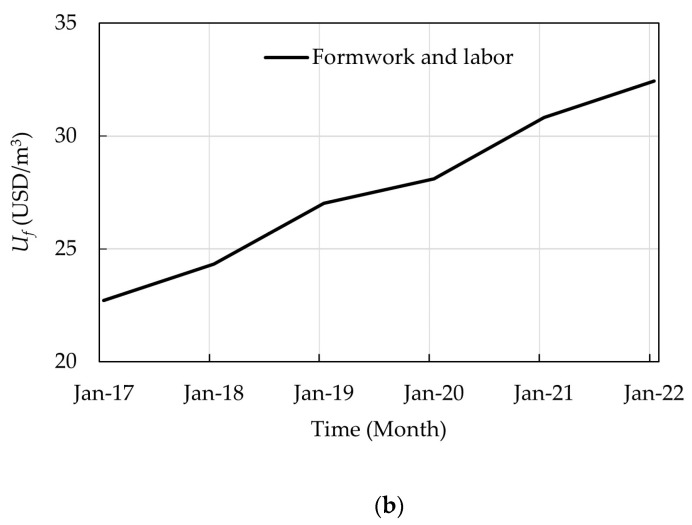
Average unit prices in the past five years (2017–2021) in Egypt: (**a**) concrete and steel reinforcement; (**b**) formwork and labor.

In practice, the concrete cost is calculated by multiplying the concrete volume $V_{c}$ by the unit price of concrete $U_{c}$, which is based on the concrete grade. The steel reinforcement cost is calculated by multiplying the steel reinforcement weight $W_{s}$ by the unit price of steel reinforcement $U_{s}$. In Egypt, the cost of formwork and labor is calculated by multiplying the concrete volume $V_{c}$ by the unit price of the formwork and labor $U_{f}$. Here, $U_{f}$ was assumed to be the same for all structural floor systems. To illustrate one of the features of the optimization problem, the unit prices $U_{c}$, $U_{s}$, and $U_{f}$ in the past five years (2017–2021) are depicted in Figure 7. The unit prices $U_{c}$ and $U_{s}$ presented in Figure 7a were obtained from the monthly bulletins of average retail prices of major important building materials provided by the Ministry of Housing, Utilities, and Urban Communities in Egypt. The unit price $U_{f}$ presented in Figure 7b represents the average yearly unit prices and was obtained from several construction sites in Egypt. It can be observed that the unit prices of materials have been fluctuating inconsistently. For instance, $U_{s}$ increased from 516 USD/ton to 746 USD/ton (45% increase) in 3 months. The unit price $U_{c}$ remained constant (39 USD/m^3^) for 17 months before increasing to 46 USD/m^3^ in December 2021. The unit price $U_{f}$ increased linearly. The fluctuation of the unit prices may change the shape of the optimization problem and, consequently, affect the best design results. Table 2 summarizes the considered unit prices.

## 4. Statement of the Problem

### 4.1. Design Variables

**Figure 8:**
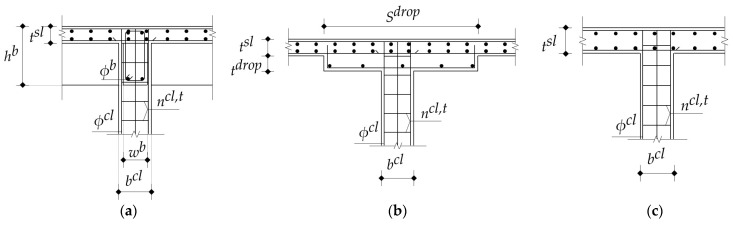
Design variables regarding concrete dimensions and steel reinforcement of each structural floor system: (**a**) SS; (**b**) FSDP; (**c**) FS.

The design variables of the optimization problem are defined in Table 3 and Figure 8 for each structural floor system, including the concrete grades, column spacings, concrete dimensions, and steel reinforcement. The concrete grades utilized in the current study were restricted to those available in the ready-mix plants in Egypt. The column spacings were between 3 m and 8 m to cover the most common spans in a building. The concrete dimensions were rounded to define increments based on the formwork dimensions to fulfill the construction requirements. The bar diameters were chosen from Egypt’s commercial steel bar list.

### 4.2. Objective Function

The general formulation of the design optimization problem can be expressed as

(81) $$\mathrm{Minimize}\;f\left(x\right){=U}_{c}V_{c}{+U}_{s}W_{s}{+U}_{f}V_{c}$$

subject to

(82) $$G_{i}^{\mathrm{Str},\;\mathrm{sl}}\left(x\right)\;\leq \;1;\;i=1,\;2,\;\ldots ,\;I$$

(83) $$G_{j}^{\mathrm{Str},\;b}\left(x\right)\;\leq \;1;\;j=1,\;2,\;\ldots ,\;J;\;b=1,\;2,\;\ldots {,\;n}_{b}$$

(84) $$G_{k}^{\mathrm{Str},\;\mathrm{cl}}\left(x\right)\;\leq \;1;\;k=1,\;2,\;\ldots ,\;K;\;\mathrm{cl}=1,\;2,\;\ldots {,\;n}_{\mathrm{cl}}$$

(85) $$G_{l}^{\mathrm{Ser},\;\mathrm{sl}}\left(x\right)\;\leq \;1;\;l=1,\;2,\;\ldots ,\;L$$

(86) $$G_{m}^{\mathrm{Ser},\;b}\left(x\right)\;\leq \;1;\;m=1,\;2,\;\ldots ,\;M;\;b=1,\;2,\;\ldots {,\;n}_{b}$$

(87) $$G_{o}^{\mathrm{Ser},\;\mathrm{cl}}\left(x\right)\;\leq \;1;\;o=1,\;2,\;\ldots ,\;O;\;\mathrm{cl}=1,\;2,\;\ldots {,\;n}_{\mathrm{cl}}$$

(88) $$x^{l}\;\leq \;x\;\leq {\;x}^{u}$$

where ***x*** is the vector of design variables; f(x) is the objective function; $G_{i}^{\mathrm{Str},\;\mathrm{sl}}\left(x\right)$, $G_{j}^{\mathrm{Str},\;b}\left(x\right)$, and $G_{k}^{\mathrm{Str},\;\mathrm{cl}}\left(x\right)$ are the strength inequality constraint functions of slabs, beams, and columns, respectively; $G_{l}^{\mathrm{Ser},\;\mathrm{sl}}\left(x\right)$, $G_{m}^{\mathrm{Ser},\;b}\left(x\right)$, and $G_{o}^{\mathrm{Ser},\;\mathrm{cl}}\left(x\right)$ are the strength inequality constraint functions of slabs, beams, and columns, respectively; *I*, *J*, and *K* are the number of strength constraints regarding slabs, beams, and columns, respectively; *L*, *M*, and *O* are the number of serviceability constraints regarding slabs, beams, and columns, respectively; $x^{l}$ and $x^{u}$ are the lower and upper bounds of the design variable ***x***; and *Z* is the number of design variables. The strength and serviceability criteria were illustrated in Section 2, while the lower and upper bounds of the design variables were presented in Table 3.

## 5. Optimization Algorithm

The mathematical model was built using Microsoft Excel 2016 spreadsheets. The model was fully programmed using Visual Basic for Applications (VBA) embedded within Microsoft Excel. Repetitive tasks, such as running the solver tool and constructing tables, were also programmed using VBA. In the current study, each structural floor system had its spreadsheet comprising all the data. This data included the calculations regarding structural analysis; design steps of structural elements; and the construction costs of concrete, steel reinforcement, and formwork and labor.

The evolutionary method was utilized by the solver tool available in Microsoft Excel to perform the optimization for two reasons. First, it uses a variety of algorithms, along with local search methods. It relies on controlled sampling combined with deterministic methods to explore the search space efficiently. Second, it can handle non-smooth and discontinuous functions [37]. The evolutionary method parameters were the population size, random seed, mutation rate, convergence value, and maximum time without improvement. Table 4 lists the values of the solver parameters used in the model.

## 6. Benchmark Examples and Discussions

**Figure 9:**
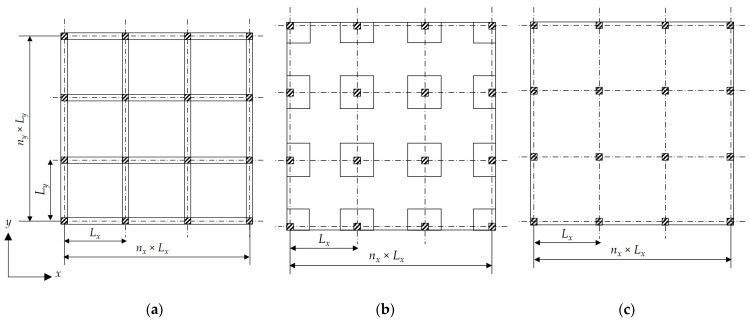
Typical floor layouts of each structural floor system: (**a**) SS; (**b**) FSDP; (**c**) FS.

Two benchmark examples with rectangular layouts were optimized (Figure 9). In the first example, two cases were examined to investigate the effects of optimizing the concrete grade and column spacings on the optimal results. The second example investigated the effects of optimizing the columns in the higher stories. The design input data of these examples are listed in Table 5.

### 6.1. Example 1: A Four-Story Building

A four-story building with a rectangular layout and a 3.3 m typical story height was considered. The total lengths of the building in the x- and y-directions were 30 m and 25 m, respectively. In case 1, the concrete grade and column spacing in the long and short directions were constant ($f_{\mathrm{cu}}$ = 35 MPa, $n_{x}$ = 5, and $n_{y}$ = 5). Here, the design variables were the parameters regarding concrete dimensions and steel reinforcement listed in Table 3. In case 2, the concrete grade and floor spacing in each direction were included in the design variables (i.e., the design variables were all the parameters defined in Table 3).

**Figure 10:**
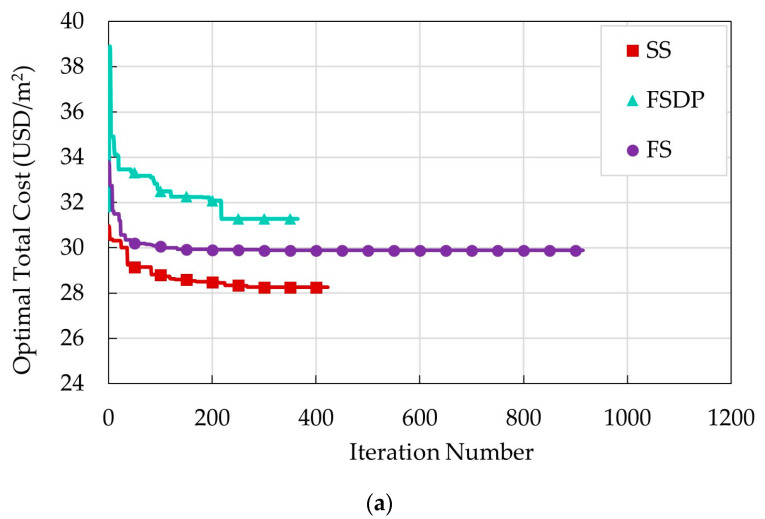

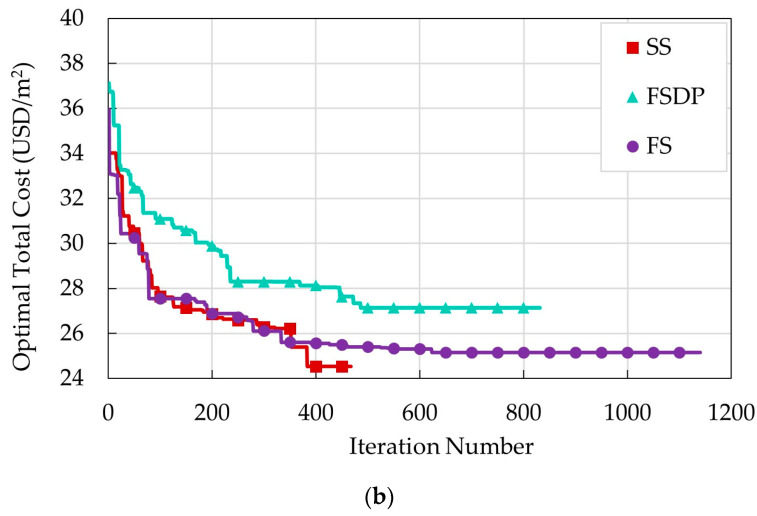
Convergence history of the best optimal runs of example 1: (**a**) case 1; (**b**) case 2.

Five runs were performed to obtain the best optimal solution. During the optimization process, the convergence history of each run was recorded. The convergence history of the best run for each structural floor system is illustrated in Figure 10. The number of iterations of each run was based on the time termination criteria, i.e., maximum time without improvement. The design variables and optimal costs of the best run of each structural floor system are displayed in Table 6 and Table 7. These results are discussed in four sections.

#### 6.1.1. Effects of Optimizing the Concrete Grade and Column Spacings

This section compares the optimal design results of case 1 and case 2 for each floor system. For all floor systems, the optimal column spacings in case 2 were smaller than the constant column spacings in case 1. Decreasing the column spacings reduced the straining actions (i.e., flexure, shear, and compressive axial loads) and deflections of structural elements. Accordingly, smaller $f_{\mathrm{cu}}$ and concrete dimensions and less steel reinforcement were sufficient to satisfy the design requirements of the structural components in case 2. Hence, the construction costs of floors decreased when the concrete grade and column spacings were optimized. As the column spacings decreased in case 2, the number of columns increased, and the concrete dimensions and steel reinforcement of columns decreased. Consequently, the columns in case 2 were more expensive than those in case 1 due to the increased number of columns. The total construction costs in case 2 were 13.2%, 16.7%, and 15.8% less than those of case 1 for SS, FSDP, and FS, respectively.

#### 6.1.2. Comparison between Floor Systems

In both cases, SS was the cheapest and FSDP was the most expensive structural floor system. The slabs of FSDP and FS utilized more steel reinforcement than those of SS slabs to satisfy the shrinkage provisions imposed, as $t^{\mathrm{sl}}$ was greater than or equal to 160 mm. Accordingly, the floors construction costs of FSDP and FS were higher than those of solid slabs.

Enhancing the slabs with drop panels reduced the punching stresses at the slab–column connections. Despite the reduction in punching stresses in FSDP compared to FS, $t^{\mathrm{sl}}$ results for FSDP and FS were the same in each case. In case 1, a high $t^{\mathrm{sl}}$ (200 mm) for FSDP was imposed to satisfy the long-term deflection criteria resulting from the heavy partition wall loads. In case 2, $t^{\mathrm{sl}}$ could not be less than the absolute minimum value (160 mm). Consequently, the floors construction costs of FSDP were higher than those of FS due to the presence of drop panels.

In case 1, SS was 9.7% and 5.5% cheaper than FSDP and FS, respectively, and FS was 4.5% cheaper than FSDP. In case 2, SS was 5.9% cheaper than FSDP and 2.5% cheaper than FSDP, and FS was 3.5% cheaper than FSDP.

#### 6.1.3. Distribution of Structural Elements Construction Costs

**Figure 11:**
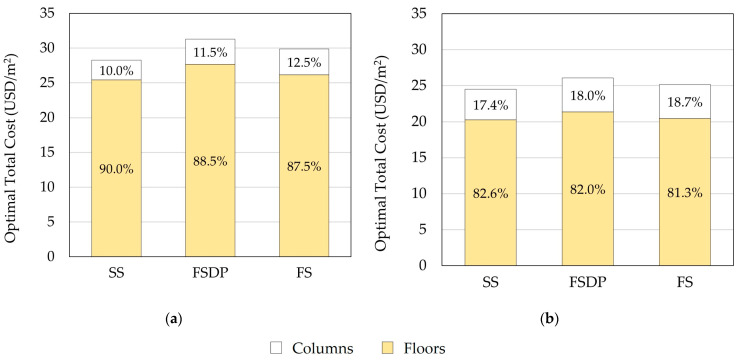
Comparison of the optimal construction total costs of floors and columns (example 1): (**a**) case 1; (**b**) case 2.

Figure 11 compares the construction costs of floors and columns for each structural floor system. In both cases, the costs of floors constituted the major part of the total construction costs. Therefore, more attention shall be paid to the optimization of floors. A similar conclusion was reported by other researchers [30,31]. In case 2, the cost ratio of columns to floors increased as a result of the increased number of columns.

#### 6.1.4. Distribution of Materials and Labor Construction Costs

**Figure 12:**
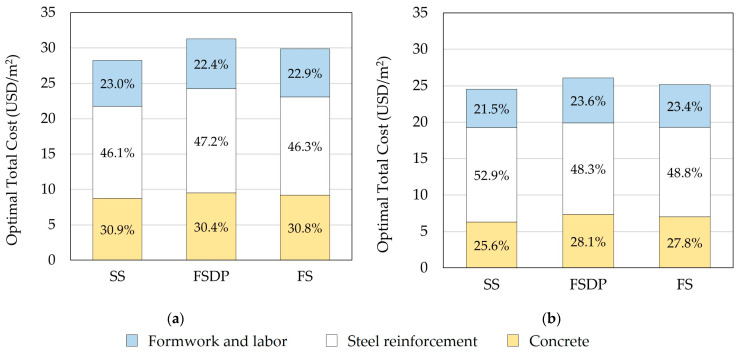
Comparison of the optimal construction total costs of materials and labor (example 1): (**a**) case 1; (**b**) case 2.

Figure 12 compares the construction costs of materials and labor for each structural floor system. In both cases, the steel reinforcement costs constituted about half the total construction costs as a result of the high unit price of steel reinforcement $U_{s}$ in Egypt. In contrast, Sahab et al. [30] reported that the steel reinforcement costs constituted the lowest construction costs. It should be mentioned that Sahab et al. [30] used different unit prices of materials and labor in accordance with Spon’s Architects’ and Builders’ Price Book 2001 [38] and Harris [39] in London, UK. Thus, the optimal design results may significantly vary based on the considered unit prices in a specific country.

### 6.2. Example 2: A Ten-Story Building

A ten-story building with a rectangular layout and a 3 m typical story height was considered. The total lengths of the building in the x- and y-directions were 35 m and 40 m, respectively. The design variables were all the parameters defined in Table 3. In this example, the concrete dimensions and steel reinforcement of columns were adjusted at three levels, namely, stories 1–4, stories 5–8, and stories 9–10, to reduce the columns’ construction costs. The characteristic strength $f_{\mathrm{cu}}$ was considered as a design variable to achieve higher cost savings of columns, as recommended by Boscardin et al. [33].

The design variables of the best run of each structural floor system are presented in Table 8 and Table 9. In this example, a higher $f_{\mathrm{cu}}$ was utilized to enhance the axial resistance of columns and reduce the long-term deflections of slabs and beams. Because the high $f_{\mathrm{cu}}$ enhanced the punching shear resistance at slab–column connections, a low $t^{\mathrm{sl}}$ (160 mm) was sufficient to resist the punching shear stresses of FSDP and FS.

The concrete dimensions of SS columns were smaller than those of FSDP and FS. This can be explained in terms of the lower axial loads and lack of punching shear stresses and bending moments at the slab–column connections of SS. At lower story levels, the concrete dimensions of the FSDP columns were larger than those of FS because the chosen $f_{\mathrm{cu}}$ for FSDP was less than that of FS.

For all structural floor systems, the optimal concrete dimensions of columns decreased at higher story levels in proportion to the reduced axial loads. However, although the axial loads applied on columns decrease at higher story levels, it can be observed that the concrete dimensions of FS columns did not decrease at stories 9–10. The lower concrete dimensions of FS columns could not resist the punching stresses.

**Figure 13:**
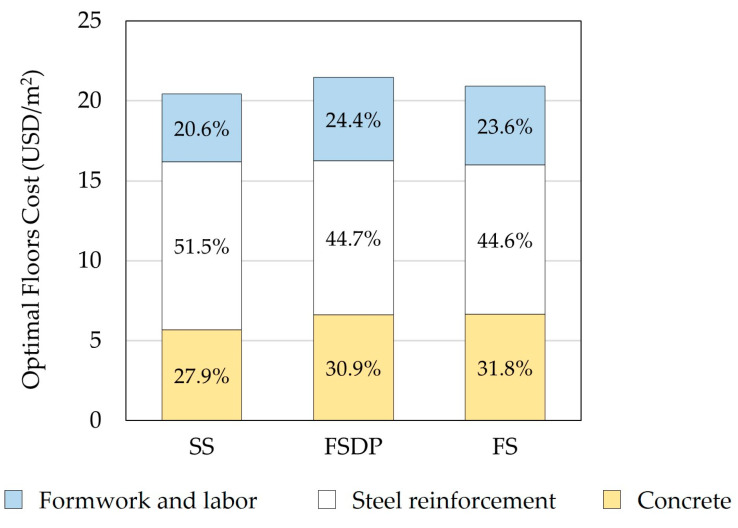
Comparison of the optimal construction costs of floors (example 2).

Figure 13 compares the optimal construction costs of the floors for a typical story for each floor system. In terms of the floors construction costs, SS was the cheapest, and FSDP was the most expensive structural floor system.

**Figure 14:**
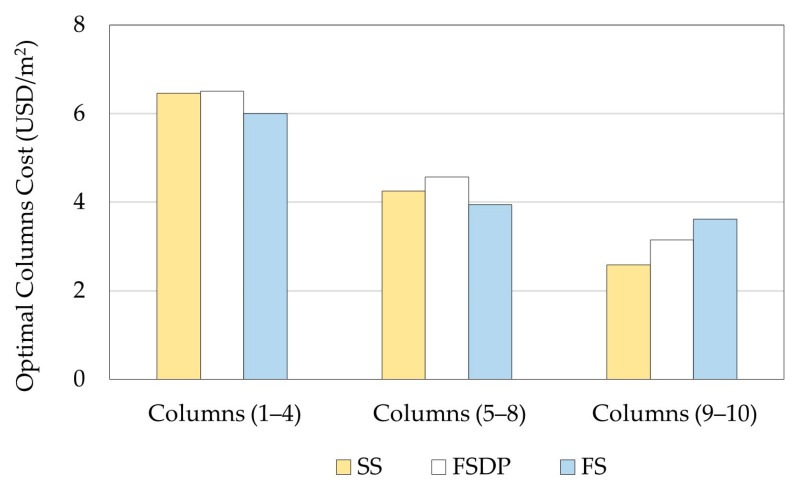
Comparison of the optimal construction costs of columns at different story levels (example 2).

Figure 14 compares the optimal construction costs of columns at different story levels for each structural floor system. As the number of stories increased, the columns cost reduction in SS was high compared to the other floor systems. It was verified that reducing the concrete dimensions of columns at higher story levels could achieve high cost savings, as reported by Boscardin et al. [33]. The total construction costs per building unit area of SS, FSDP, and FS were 25.21 USD/m^2^, 26.52 USD/m^2^, and 25.57 USD/m^2^, respectively. Hence, SS was 4.9% and 1.6% cheaper than FSDP and FS, respectively, and FS was 3.4% cheaper than FSDP.

## 7. Conclusions

In this study, a design optimization model was developed to minimize the construction costs of materials and labor in RC buildings. The optimization model was built using the evolutionary algorithm provided by the Microsoft Excel solver tool. Three structural floor systems were considered: SS, FSDP, and FS. The design constraints were based on the design provisions of ECP 203-18 to ensure the safety of each floor system. Discrete values of design variables were utilized per construction industry requirements to account for the practical considerations.

Two benchmark examples were considered to investigate the effects of the design variables on the optimal results of each structural floor system. The results revealed that solid slab buildings produced the most cost savings among the three systems.

The construction costs could be significantly reduced by considering the concrete compressive strength and the column spacings as design variables. The optimizer tended to reduce the column spacings to minimize the straining actions of the structural elements and, consequently, achieve economical concrete dimensions and steel reinforcement.

Low concrete grades are sufficient for low-rise residential buildings with small column spacings to resist the low stresses applied on floors and columns. As the number of stories increases, the tendency toward selecting a higher concrete grade increases to reduce the concrete dimensions of columns. The cost of columns decreased significantly with reducing the concrete dimensions in the higher story levels. In the case of FS, the cost of columns was less likely to decrease in higher story levels to resist the punching shear stresses. The cost of floors constituted the major part of the construction cost concerning the structural elements.

The optimal distribution of construction materials can vary based on the considered unit prices. In the current study, steel reinforcement constituted about 50% of the construction cost due to the high unit price of steel reinforcement in Egypt.

The current study revealed the possibilities of determining an economical structural floor system, concrete grade, concrete dimensions, and steel reinforcement of RC buildings by considering the integration of structural components. The contribution of the concrete grade and column spacing in reducing the construction costs was discussed. The presented optimization method could be applied to RC buildings subjected to seismic and wind loads in future work by considering the additional load cases provided by the design code. Thus, the effects of the additional design constraints on the optimal construction costs could be examined. The input data, design calculations, and unit costs of materials and labor could be adjusted to fulfill the restrictions of any design code or consider different structural floor systems.

## Figures and Tables

**Table 1:** Critical shear parameters at the slab–column connections.

Type of Column	Interior	Edge	Corner
**Shape**	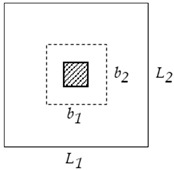	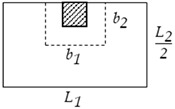	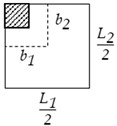
**Critical shear length** $b_{1}$	$t^{\mathrm{cl}}{+d}_{p}$	$t^{\mathrm{cl}}{+d}_{p}$	$t^{\mathrm{cl}}+\frac{d_{p}}{2}$
**Critical shear width** $b_{2}$	$b^{\mathrm{cl}}{+d}_{p}$	$b^{\mathrm{cl}}+\frac{d_{p}}{2}$	$b^{\mathrm{cl}}+\frac{d_{p}}{2}$
**Critical shear perimeter** $p^{\mathrm{cl}}$	*2* $\left(b_{1}{+b}_{2}\right)$	$b_{1}{+2b}_{2}$	$b_{1}{+b}_{2}$
**Critical shear area** $A^{\mathrm{cl},\mathrm{cr}}$	$b_{1}b_{2}$	$b_{1}b_{2}$	$b_{1}b_{2}$
**Tributary area** $A^{\mathrm{cl},\mathrm{tr}}$	$L_{1}L_{2}$	$\frac{L_{1}L_{2}}{2}$	$\frac{L_{1}L_{2}}{4}$

**Table 2:** Unit prices of the cost components.

	Component	Strength (MPa)	Unit	Price (USD/Unit)
$U_{c}$	Concrete	25	m^3^	36.8
		30		39.2
		35		41.6
		40		44.1
		45		48.4
		50		52.7
		55		57.0
		60		61.4
$U_{s}$	High tensile steel	420	ton	735.1
	Mild steel	240		735.1
$U_{f}$	Formwork and labor	-	m^3^	30.8

**Table 3:** Design variables of different structural floor systems.

Parameter	Design Variable	Symbol	Increment/Set	Lower Bound	Upper Bound	SS	FSDP	FS
Concrete grade	Characteristic compressive strength	$f_{\mathrm{cu}}$	{25, 30, 35, 40, 45, 50, 55, 60} MPa	25 MPa	60 MPa	*✓*	*✓*	*✓*
Column spacings	Number of spans (x-direction)	$n_{x}$	1	$\max\left(L_{x}/8000\;\mathrm{mm},\;3\right)$	$L_{x}/3000\;\mathrm{mm}$	*✓*	*✓*	*✓*
	Number of spans (y-direction)	$n_{y}$		$\max\left(L_{y}/8000\;\mathrm{mm},\;3\right)$	$L_{y}/3000\;\mathrm{mm}$	*✓*	*✓*	*✓*
Concrete dimensions	Slab thickness	$t^{\mathrm{sl}}$	20 mm	$\max\left(L_{2}/40,\;80\;\mathrm{mm}\right)\;\mathrm{for}\;\mathrm{SS}$ $\max\left(L_{1}/36,\;150\;\mathrm{mm}\right)\;\mathrm{for}\;\mathrm{FSDP}$ $\max\left(L_{1}/32,\;150\;\mathrm{mm}\right)\;\mathrm{for}\;\mathrm{FS}$	300 mm	*✓*	*✓*	*✓*
	Beam height	$h^{b}$	50 mm	$\max\left({3t}^{\mathrm{sl}},\;400\;\mathrm{mm}\right)$	900 mm	✓	-	-
	Beam width	$w^{b}$	50 mm	$\max\left(b_{w},\;250\;\mathrm{mm}\right)$	400 mm	✓	-	-
	Drop panel thickness	$t^{\mathrm{drop}}$	20 mm	$t^{\mathrm{sl}}/4$	120 mm	-	*✓*	-
	Drop panel width	$S^{\mathrm{drop}}$	50 mm	$L_{1}/3$	$L_{2}/2$	-	*✓*	-
	Interior column width	$b^{\mathrm{in}}$	50 mm	250 mm for SS $\max\left(h/{15,\;L}_{1}/20,\;300\;\mathrm{mm}\right)\;\mathrm{for}\;\mathrm{FSDP}$ $\max\left(h/{15,\;L}_{1}/20,\;300\;\mathrm{mm}\right)\;\mathrm{for}\;\mathrm{FS}$	800 mm	*✓*	*✓*	*✓*
	Edge column width (x-direction)	$b^{\mathrm{ex}}$				*✓*	*✓*	*✓*
	Edge column width (y-direction)	$b^{\mathrm{ey}}$				*✓*	*✓*	*✓*
	Corner column width	$b^{\mathrm{cr}}$				*✓*	*✓*	*✓*
Steel reinforcement	Beam bar diameter	$\phi^{b}$	{10, 12, 16, 18, 22, 25} mm	10 mm	25 mm	*✓*	-	-
	Interior column bar diameter	$\phi^{\mathrm{in}}$	{12, 16, 18, 22, 25, 28} mm	12 mm	28 mm	*✓*	*✓*	*✓*
	Edge column bar diameter (x-direction)	$\phi^{\mathrm{ex}}$				*✓*	*✓*	*✓*
	Edge column bar diameter (y-direction)	$\phi^{\mathrm{ey}}$				*✓*	*✓*	*✓*
	Corner column bar diameter	$\phi^{\mathrm{cr}}$				*✓*	*✓*	*✓*
	Number of interior column lateral ties	$n^{\mathrm{in},t}$	1	$\max\left({1000t}^{\mathrm{sl}}/15\phi^{\mathrm{in}},\;5\right)$	10	*✓*	*✓*	*✓*
	Number of edge column lateral ties (x-direction)	$n^{\mathrm{ex},t}$		$\max\left({1000t}^{\mathrm{sl}}/15\phi^{\mathrm{ex}},\;5\right)$		*✓*	*✓*	*✓*
	Number of edge column lateral ties (y-direction)	$n^{\mathrm{ey},t}$		$\max\left({1000t}^{\mathrm{sl}}/15\phi^{\mathrm{ey}},\;5\right)$		*✓*	*✓*	*✓*
	Number of corner column lateral ties	$n^{\mathrm{cr},t}$		$\max\left({1000t}^{\mathrm{sl}}/15\phi^{\mathrm{cr}},\;5\right)$		*✓*	*✓*	*✓*

**Table 4:** Microsoft Excel solver parameters.

Parameter	Value
Constraint precision	1 × 10^−6^
Maximum time	Unrestricted
Iterations	Unrestricted
Maximum subproblems	Unrestricted
Maximum feasible solutions	Unrestricted
Convergence	1 × 10^−4^
Mutation rate	0.075
Population size	100
Random seed	0
Maximum time without improvement	120 s

**Table 5:** Design input data.

Parameter		Value
$f_{y}$	Yield strength of the longitudinal steel reinforcement	420 MPa
$f_{y,\mathrm{st}}$	Yield strength of the lateral steel reinforcement	240 MPa
$E_{s}$	Elastic modulus of steel	200 GPa
$\gamma_{\mathrm{rc}}$	Unit weight of concrete	25 kN/m^3^
$\gamma_{\mathrm{st}}$	Unit weight of steel	78.5 kN/m^3^
$\gamma_{b}$	Unit weight of brick partition walls	14 kN/m^3^
$\gamma_{c}$	Safety reduction factor for concrete	1.5
$\gamma_{s}$	Safety reduction factor for steel	1.15
$c^{\mathrm{sl}}$	Concrete cover of slabs	25 mm
$c^{b}$	Concrete cover of beams	50 mm
$c^{\mathrm{cl}}$	Concrete cover of columns	25 mm
*p*	Live load	2 kPa
$w_{f}$	Flooring load	1.5 kPa
$\phi_{\mathrm{st}}$	Bar diameter of lateral steel reinforcement	8 mm

**Table 6:** Summary of the optimal concrete grade, column spacings, concrete dimensions of floors, and costs of floors (example 1).

Floor System	Case	$n_{x}{\;\times \;n}_{y}$	$L_{x}{\;\times \;L}_{y}$ (mm)	$f_{\mathrm{cu}}$ (MPa)	$t^{\mathrm{sl}}$ (mm)	$t^{\mathrm{drop}}$ (mm)	$S^{\mathrm{drop}}$ (mm)	$h^{b}$ (mm)	$w^{b}$ (mm)	Floors Cost (USD/m^2^)
SS	1	5 × 5	6000 × 5000	35	140	-	-	500	250	25.43
	2	8 × 8	3750 × 3125	25	80	-	-	400	250	20.25
FSDP	1	5 × 5	6000 × 5000	35	200	60	2000	-	-	27.66
	2	8 × 8	3750 × 3125	25	160	40	1600	-	-	21.36
FS	1	5 × 5	6000 × 5000	35	200	-	-	-	-	26.16
	2	8 × 8	3750 × 3125	25	160	-	-	-	-	20.46

**Table 7:** Summary of the optimal concrete dimensions, steel reinforcement, and costs of columns (example 1).

Floor System	Case	Interior Columns			Edge Columns (*x*-Direction)			Edge Columns (*y*-Direction)			Corner Columns			Columns Cost (USD/m^2^)
		$b^{\mathrm{in}}$ (mm)	Steel Bars	No. of Columns	$b^{\mathrm{ex}}$ (mm)	Steel Bars	No. of Columns	$b^{\mathrm{ey}}$ (mm)	Steel Bars	No. of Columns	$b^{\mathrm{cr}}$ (mm)	Steel Bars	No. of Columns	
SS	1	400	8T16	16	300	4T16	8	250	4T18	8	250	4T16	4	2.83
	2	300	4T16	49	250	4T16	14	250	4T16	14	250	4T16	4	4.28
FSDP	1	400	8T16	16	350	8T16	8	350	8T16	8	300	4T16	4	3.61
	2	300	4T16	49	300	4T16	14	300	4T16	14	300	4T16	4	4.70
FS	1	400	8T16	16	400	8T16	8	350	8T16	8	300	4T16	4	3.72
	2	300	4T16	49	300	4T16	14	300	4T16	14	300	4T16	4	4.70

**Table 8:** Summary of the optimal concrete grade, column spacings, concrete dimensions of floors, and costs of floors (example 2).

Floor System	$n_{x}{\;\times \;n}_{y}$	$L_{x}{\;\times \;L}_{y}$ (mm)	$f_{\mathrm{cu}}$ (MPa)	$t^{\mathrm{sl}}$ (mm)	$t^{\mathrm{drop}}$ (mm)	$S^{\mathrm{drop}}$ (mm)	$h^{b}$ (mm)	$w^{b}$ (mm)	Floors Cost (USD/m^2^)
SS	11 × 8	3182 × 5000	35	80	-	-	400	250	20.42
FSDP	7 × 8	5000 × 5000	30	160	40	2450	-	-	21.46
FS	7 × 8	5000 × 5000	35	160	-	-	-	-	20.91

**Table 9:** Summary of the optimal concrete dimensions, steel reinforcement, and cost of columns (example 2).

**Floor** **System**	**Stories**	Interior Columns			Edge Columns (*x*-Direction)			Edge Columns (*y*-Direction)			Corner Columns			Columns Cost (USD/m^2^)
		$b^{\mathrm{in}}$ (mm)	Steel Bars	No. of Columns	$b^{\mathrm{ex}}$ (mm)	Steel Bars	No. of Columns	$b^{\mathrm{ey}}$ (mm)	Steel Bars	No. of Columns	$b^{\mathrm{cr}}$ (mm)	Steel Bars	No. of Columns	
SS	1–4	450	8T18	70	350	8T16	14	350	8T16	20	250	4T16	4	6.45
	5–8	350	8T16		300	4T16		250	4T16		250	4T16		4.25
	9–12	250	4T16		250	4T16		250	4T16		250	4T16		2.58
FSDP	1–4	600	12T18	42	400	8T18	12	400	8T18	12	350	8T18	4	6.51
	5–8	500	8T18		350	8T16		350	8T16		300	4T18		4.57
	9–10	400	8T16		300	4T16		300	4T16		300	4T16		3.15
FS	1–4	550	8T22	42	400	8T16	12	400	8T16	12	300	4T16	4	5.99
	5–8	400	8T18		350	8T16		350	8T16		300	4T16		3.95
	9–10	400	8T16		350	8T16		350	8T16		300	4T16		3.61

## Data Availability

All data generated or used during the study are available from the corresponding author by request.
